# Edge‐Localized Plasmonic Resonances in WS_2_ Nanostructures from Electron Energy‐Loss Spectroscopy

**DOI:** 10.1002/smsc.202400558

**Published:** 2025-02-13

**Authors:** Abel Brokkelkamp, Sabrya E. van Heijst, Sonia Conesa‐Boj

**Affiliations:** ^1^ Kavli Institute of Nanoscience Delft University of Technology 2628 CJ Delft Netherlands

**Keywords:** electron energy loss spectroscopy, localized plasmonic resonances, non‐negative matrix factorization, transition metal dichalcogenides, tungsten disulfide (WS_2_)

## Abstract

Localized plasmon resonances in 2D transition metal dichalcogenides (TMDs) offer a powerful means to enhance light–matter interactions at the nanoscale, making them ideal candidates for advanced optoelectronic applications. However, disentangling the complex plasmonic interactions in these materials, especially in the low‐energy regime, presents significant challenges. Herein, localized plasmon resonances in chemical vapor deposition‐grown tungsten disulfide (WS_2_) nanotriangles, using a combination of advanced spectral analysis and simulation techniques, is investigated. By combining non‐negative matrix factorization with electron energy loss spectroscopy, distinct plasmonic modes to provide a comprehensive analysis of the plasmonic landscape of individual and stacked WS_2_ nanotriangles are identified and characterized. Furthermore, the dispersion relation of these localized plasmon resonances is quantified and their evolution across different WS_2_ triangular geometries is evaluated. Experimental characterization of plasmonic resonances in WS_2_ through dedicated numerical simulations based on the pygdm package is validated. The findings highlight the critical role of localized plasmon resonances in modulating the electronic and optical properties of WS_2_, offering new insights into the design and optimization of TMD‐based devices for optoelectronic and nanophotonic applications.

## Graphical abstract

Localized plasmon resonances in chemical vapor deposition‐grown tungsten disulfide WS_2_ nanotriangles are investigated using electron energy‐loss spectroscopy and non‐negative matrix factorization. Distinct plasmonic modes and their dispersion relations are characterized and validated through numerical simulations, providing insights into the plasmonic landscape of WS_2_ for advanced optoelectronic and nanophotonic applications.

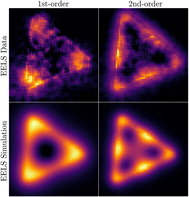

## 1 Introduction

2D transition metal dichalcogenides (TMDs) have emerged as a versatile family of materials with extraordinary optoelectronic properties,^[^ ^1^, ^2^, ^3^, ^4^, ^5^ ^]^ positioning them at the forefront of nanoscale device research. Among TMDs, tungsten disulfide (${\text{WS}}_{2}$) stands out due to its direct bandgap and strong excitonic effects,^[^ ^5^, ^6^, ^7^, ^8^, ^9^ ^]^ making it highly attractive for applications in light harvesting,^[^ ^10^, ^11^, ^12^ ^]^ sensing,^[^ ^13^, ^14^, ^15^ ^]^ and quantum optics.^[^ ^16^, ^17^, ^18^ ^]^ However, while excitonic behavior of ${\text{WS}}_{2}$ has been extensively studied, its plasmonic properties, particularly in the low‐energy regime, remain less explored.

Specifically, spatially localized plasmon resonances in 2D materials have garnered increasing interest due to their ability to confine electromagnetic fields at the nanoscale, hence enabling enhanced light–matter interactions.^[^ ^19^, ^20^, ^21^, ^22^ ^]^ In ${\text{WS}}_{2}$ nanostructures, the geometric arrangement, thickness, and edge configurations play critical roles in determining the plasmonic response, which can be tuned to optimize device performance. The ability to precisely characterize these localized plasmonic modes at the nanoscale is key to harnessing them for applications in optoelectronics and nanophotonics.^[^ ^23^, ^24^ ^]^

To this aim, electron energy‐loss spectroscopy (EELS) provides a powerful technique for probing localized plasmonic excitations,^[^ ^25^, ^26^, ^27^ ^]^ uniquely combining high spatial precision with competitive energy resolution.^[^ ^28^ ^]^ In particular, EELS enables a detailed characterization of the low‐loss region (ΔE≤50 eV) where multiple plasmonic and excitonic resonances are expected. The main challenge here lies in the interpretation of the complex spectral data, where overlapping signals make it difficult to cleanly disentangle localized plasmon resonances in dielectrics^[^ ^29^ ^]^ and fully resolving the individual modes. Tackling these challenges requires advancements in data analysis techniques, such as non‐negative matrix factorization (NMF), which have shown considerable promise in separating overlapping spectral features^[^ ^30^, ^31^, ^32^ ^]^ and unveiling hidden plasmonic resonances.

Here, we report the identification of localized plasmonic modes in ${\text{WS}}_{2}$ nanostructures using a combination of advanced EELS and NMF analyses. By applying NMF to low‐loss EELS data, we disentangle the distinct localized plasmonic components arising across various ${\text{WS}}_{2}$ triangular geometries. To further validate our findings, we demonstrate the agreement of the results with dedicated electrodynamical simulations. These numerical simulations confirm the features of the extracted localized plasmonic modes and provide complementary insights on their spatial and spectral evolution.

Our results pave the way for the enhanced design of ${\text{WS}}_{2}$‐based devices, where the precise control over the performance of localized plasmonic resonances leads to improved functionalities in next‐generation optoelectronic and nanophotonic technologies.

## 2 Results and Discussion

**Figure 1:**
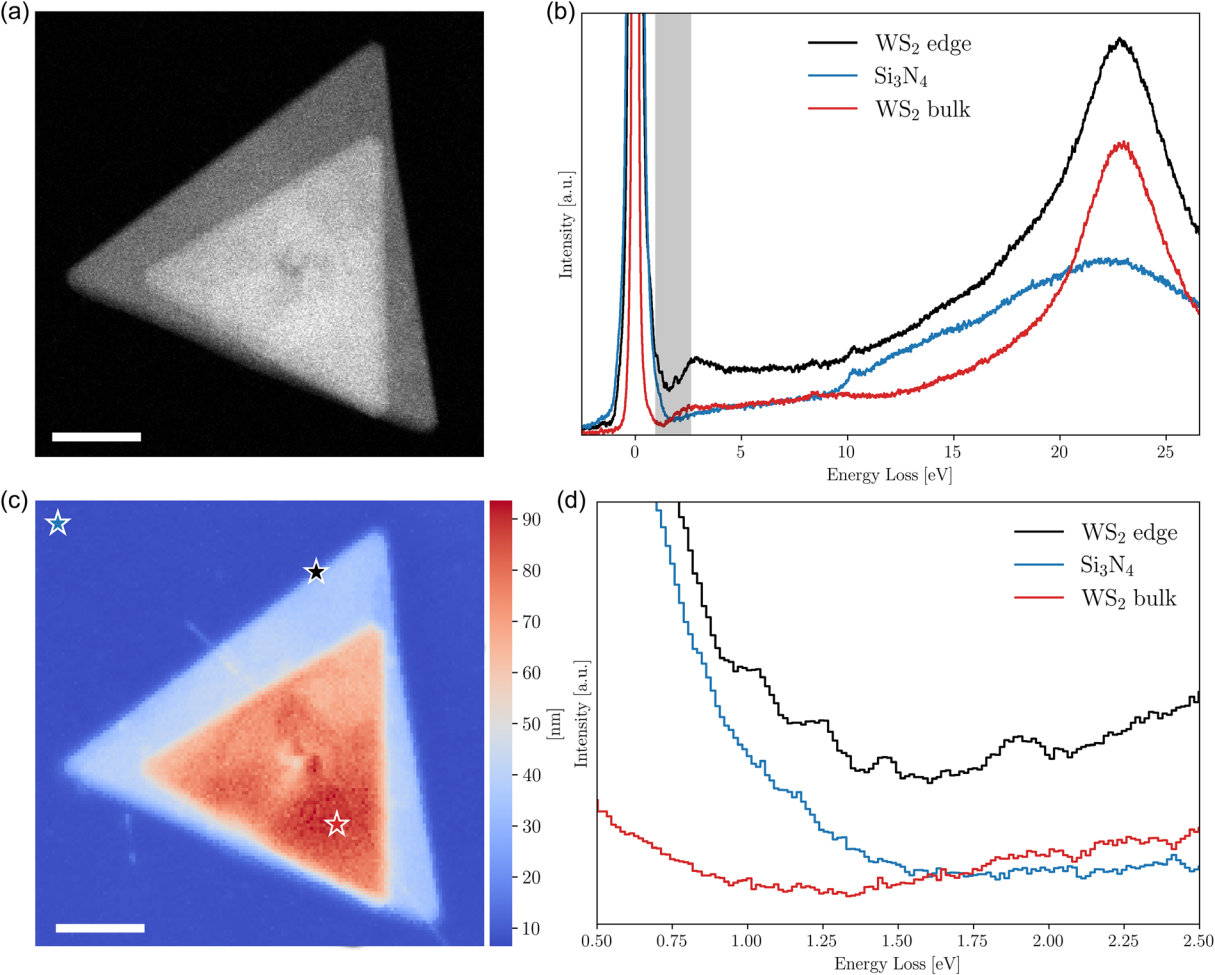
Characterization of the stacked ${\text{WS}}_{2}$ nanotriangles. a) High‐angle‐annular dark‐field STEM image of the stacked ${\text{WS}}_{2}$ nanotriangles, revealing their morphology and relative arrangement. c) The associated thickness map obtained from the EELS spectral image. Scale bars indicate 200 nm. b) Averaged EELS taken from the three regions highlighted in (c): ${\text{WS}}_{2}$ edge, ${\text{WS}}_{2}$ bulk, and Si_3_N_4_ substrate. The shaded area indicates the energy range between 0.5eV and 2.5 eV. d) Close up of the energy range highlighted in (b) and displaying, as will be shown in Figure 2, periodic oscillations corresponding to distinct plasmonic modes.

The ${\text{WS}}_{2}$ nanostructures investigated in this work were synthesized using chemical vapor deposition (CVD) and directly grown on a 5 nm‐thick ${\text{Si}}_{3}N_{4}$ membrane. This process yielded well‐defined triangular ${\text{WS}}_{2}$ morphologies, with side lengths ranging from 330 to 980 nm (see Section S1, Supporting Information). In addition to single ${\text{WS}}_{2}$ nanotriangles, more complex configurations such as the stacked ${\text{WS}}_{2}$ nanotriangles shown in **Figure** **1**a are also present. In this nanostructure, two misaligned triangles are stacked on top of each other, with the smaller triangle (side lengths 680 nm) rotated by 6° relative to the larger one at the base (side lengths 980 nm).

Both single and stacked ${\text{WS}}_{2}$ nanostructures exhibit uniform thicknesses, as verified through scanning transmission electron microscopy (STEM) and EELS measurements (see Section S1, Supporting Information). These nanostructures represent a flexible platform to investigate spatially localized ${\text{WS}}_{2}$ plasmons, with the single triangles providing the baseline and the stacked structures a complementary avenue for exploring novel plasmonic phenomena in ${\text{WS}}_{2}$. Detailed information regarding the growth methodology is provided in the Section 4.

Our investigations of the ${\text{WS}}_{2}$ specimens focus on the low‐loss EELS region, targeting energy losses ΔE≤50 eV to uncover plasmonic or excitonic signatures.^[^ ^29^, ^33^ ^]^ Representative EELS obtained from the staked nanotriangles of Figure 1a are displayed in Figure 1b, corresponding to the regions marked by the star‐shaped symbols (10×10 pixel area average) in the thickness map of Figure 1c. The chosen regions highlight the bulk ${\text{WS}}_{2}$ region, the transition zone at the ${\text{WS}}_{2}$ edges, and the surrounding ${\text{Si}}_{3}N_{4}$ membrane.^[^ ^34^ ^]^

The spectra for both the bulk ${\text{WS}}_{2}$ region and the ${\text{Si}}_{3}N_{4}$ membrane show distinct features characteristic of each material. The ${\text{Si}}_{3}N_{4}$ membrane spectrum displays, as expected, the bulk plasmon peak at 22.3 eV. This ${\text{Si}}_{3}N_{4}$ contribution appears superimposed with the spectrum of ${\text{WO}}_{3}$ used for the CVD growth process, which is indicated by the small peak at 10.2 eV and characteristic for oxides.^[^ ^35^ ^]^ The bulk ${\text{WS}}_{2}$ region exhibits a pronounced peak at 23 eV corresponding to the bulk plasmon and a peak at 8 eV associated with the surface plasmon.^[^ ^36^, ^37^ ^]^

Interestingly, in the transition region at the ${\text{WS}}_{2}$ edges, one finds sizeable new contributions to the spectrum for energy losses between the zero loss peak (ZLP) and the onset of the inelastic scattering continuum. These contributions are highlighted in gray in Figure 1b and further amplified in Figure 1d. This energy window reveals periodic oscillations spanning from 0.5 to 2.5 eV, which are absent from the spectra of either bulk ${\text{WS}}_{2}$ or ${\text{Si}}_{3}N_{4}$, suggesting the presence of low‐energy excitations localized at the ${\text{WS}}_{2}$ edges. Given that TMDs such as ${\text{WS}}_{2}$ are known to exhibit metallic‐like edge states, especially for sharp geometries,^[^ ^12^, ^35^, ^38^, ^39^ ^]^ these edge regions may provide favorable conditions for the formation of localized plasmon resonances. While surface plasmon resonances are primarily influenced by geometry and refractive index, the presence of metallic edge states in TMDs can enhance plasmonic behavior by supporting free or quasi‐free charge carriers along the edges.

### 2.1 NMF Decomposition of Spectral Features

The recorded ${\text{WS}}_{2}$ EELS spectra are composed by a complex mixture of overlapping features. To investigate the physical mechanisms underlying the periodic signals identified in the edge regions of the ${\text{WS}}_{2}$ nanotriangles between 0.5 and 2.5 eV in Figure 1, we employ two complementary EELS data processing techniques. First, a machine learning approach was deployed^[^ ^40^, ^41^, ^42^, ^43^ ^]^ for the model‐independent subtraction of the ZLP background contaminating the inelastic scattering onset region, enhancing feature significance for energy losses critical for our analysis (see Figure S4, Supporting Information). Next, NMF from scikit‐learn was applied to the ZLP‐subtracted EELS spectral image to disentangle and resolve specific features arising for specific energy‐loss windows, enabling the interpretability of the individual components to the total low‐loss spectra.

NMF is a blind‐source separation technique that can efficiently disentangle individual features present in the measured EELS spectra, enabling a refined understanding of the different plasmonic and excitonic resonances in a material. As such, it has found widespread use in EELS analyses of, for example, metallic nanostructures.^[^ ^44^, ^45^, ^46^, ^47^, ^48^ ^]^ Similarly to the popular principal component analysis method, NMF also ranks the identified components by their statistical significance, starting from the most dominant feature. To reduce statistical noise while preserving spatial resolution, we applied pooling with a 2D Gaussian profile before performing the NMF decomposition to the complete EELS spectral image. Further details about the NMF decomposition of the ${\text{WS}}_{2}$ nanostructures are provided in the Section S2.2, Supporting Information.

**Figure 2:**
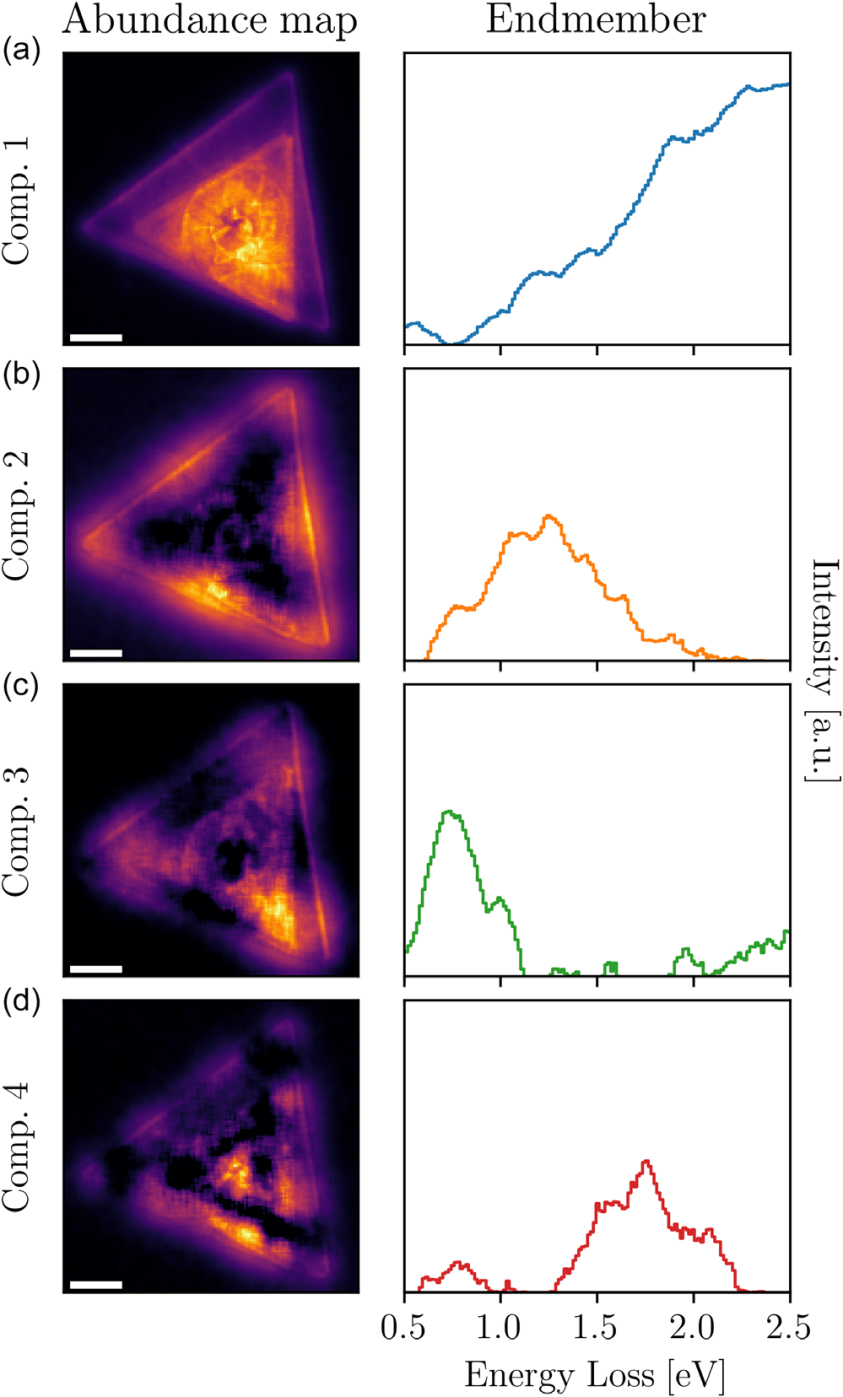
NMF decomposition of EELS in stacked ${\text{WS}}_{2}$ nanotriangles. a–d) The abundance maps (left) and endmembers (right panels) of the first four components of the NMF decomposition of the stacked ${\text{WS}}_{2}$ nanotriangle. Component 1 represents the bulk ${\text{WS}}_{2}$ spectrum, with the A‐ and B‐exciton peaks at 1.9 and 2.3 eV. Component 2 corresponds to the second‐order resonance mode centered around 1.25 eV, with the abundance map displaying its characteristic node structure. Components 3 and 4 are dominated by the first‐order and third‐order resonance modes of the large triangle, peaking at 0.75 and 1.75 eV, respectively. In the right panels, vertical axes are uniformly scaled. Scale bars represent 200 nm.

**Figure** **2** displays the result of applying NMF to the EELS spectral image associated with ${\text{WS}}_{2}$ stacked nanotriangles specimen of Figure 1 when restricted to the energy window of interest, namely [0.5,2.5] eV. The outcome of a NMF decomposition is given by two elements for each components: the endmember, which corresponds to the identified unique spectral feature, and an abundance map, indicating the spatial distribution of the corresponding endmember across the EELS spectral image. In Figure 2 we limit ourselves to the first four components, which represent a cumulative variance of around 98.5% as shown in the Figure S4c, Supporting Information. Although endmembers are defined as pure signals, in some cases, a given component may still reflect a superposition of multiple signals exhibiting overlapping features or abundances. In the particular case of dielectric materials, it has been shown that their resonance modes are less defined as compared to their counterparts in metals,^[^ ^29^ ^]^ which can lead to features having a residual contribution to other NMF components beyond the main associated one.

Inspection of the NMF components reported in Figure 2, together with analogous studies for individual ${\text{WS}}_{2}$ nanotriangles, reveals localized plasmon resonances arising in both the single and stacked triangular configurations. In the latter case, resonance modes in the two overlapping triangles may overlap in both energy loss and spatial localization, which together with the worse definition of spectral features in dielectric materials demands a careful interpretation of the identified components.

The NMF component 1 in Figure 2 corresponds to the bulk ${\text{WS}}_{2}$ spectrum. The characteristic A and B exciton peaks at the expected values around 1.9 and 2.3 eV are observed. Additional features at ≈1.2 and 1.4 eV can also be seen. Though their origin remains unclear, their presence in component 1 suggests they are features related to the crystal structure, in particular to the surface of the smaller nanotriangle which may exhibit structural defects and strain.^[^ ^49^ ^]^

NMF components 2–4 in Figure 2 provide direct insights into the nature of the localized plasmonic behavior of the inspected ${\text{WS}}_{2}$ nanostructure, with each component revealing different features which would otherwise remain hidden in the EELS spectral image due to overlapping signals. The abundance map associated with component 2 clearly reveals its association with the second‐order plasmonic resonance localized at the edges of the larger triangle, with three maxima and two minima across each edge. The corresponding endmember reveals a broad peak around 1.25 eV together with additional weaker signals. Component 3 displays two distinct peaks at 0.75 and 1 eV, which, based on the abundance map, are associated with the first‐order resonances of both the large and small triangle, as confirmed by the dedicated theoretical EELS simulations to be discussed in Figure 4 and Figure S14, Supporting Information. The overlap in their spatial localization at the corners of the triangles likely causes the two features to appear associated with the same component. Finally, component 4 features a broad peak centered around 1.75 eV, whose periodicity indicates the association with the third‐order resonance in large triangle. We compare the components to point spectra selected from regions of high and low abundance in the abundance maps (see Figure S10, Supporting Information) and confirm the presence/absence of these features depending on the high/low abundance.

The NMF decomposition of Figure 2 is restricted to the [0.5,2.5] eV and finds first‐, second‐, and third‐order resonance modes. By extending the analysis to a broader energy window to [2.0,4.0] eV, it is possible to identify higher‐order modes, as demonstrated in the Figure S6, Supporting Information. Specifically, we detect fourth‐ and fifth‐order resonance modes for the large triangle, as well as indications for third‐ and fourth‐order resonance modes in the small triangle.

The same NMF analysis has also been applied to standalone ${\text{WS}}_{2}$ nanotriangles with side lengths of 330, 880, and 920 nm single nanotriangles. For the 880 and 920 nm nanotriangles, we uncover the first‐, second‐, and third‐order resonance modes in the [0.5,2.5] eV energy window (Figure S9 and S7, Supporting Information, respectively). For the 330 nm nanotriangle, an energy window of [0.5,4.0] eV reveals the first‐ and second‐order resonance modes (Figure S8, Supporting Information). All in all, the emerging picture of localized low‐energy plasmonic resonances in ${\text{WS}}_{2}$ is fully consistent between single and stacked nanotriangles.

### 2.2 Electrodynamical Simulations of EELS Edge Profiles

To validate the plasmonic interpretation of the localized NMF components identified in the ${\text{WS}}_{2}$ nanostructures in Figure 2, we have carried out dedicated electrodynamical simulations. Specifically, we numerically simulate the expected energy loss of the STEM–EELS electrons interacting with the edges of the large ${\text{WS}}_{2}$ nanotriangle using the pygdm Python package,^[^ ^50^, ^51^ ^]^ denoted as edge profiles in the following. This package can be used to solve Maxwell's equations for arbitrary geometries and is based on a discretization technique, the Green dyadic method (GDM), together with a generalized propagator for fast simulations. Here we simulate the EELS edge profiles of our ${\text{WS}}_{2}$ geometries using the fast electron module of pygdm, with the incident electrons impinging at 200 keV perpendicular to ${\text{WS}}_{2}$ specimen. The simulations account for the experimental conditions with the ${\text{WS}}_{2}$ nanotriangles on top of a ${\text{Si}}_{3}N_{4}$ substrate surrounded by vacuum. Optical constants for ${\text{WS}}_{2}$ were taken from refractiveindex.info.^[^ ^52^ ^]^

**Figure 3:**
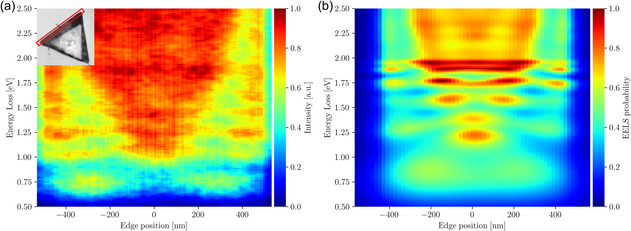
Experimental and predicted EELS edge profiles in ${\text{WS}}_{2}$ nanotriangles. a) Experimental EELS edge profile (indicated in the inset, length of 980 nm) of the stacked ${\text{WS}}_{2}$ nanotriangles of Figure 1. b) The predictions of the corresponding electrodynamical simulation based on the pygdm package. Both maps are normalized to their maximum value. Qualitative agreement in the energy‐loss region of interest [0.5,2.5] eV is observed, in particular concerning the oscillatory resonance structure characteristic of the identified plasmonic edge modes. The marked enhancement of the EELS edge profiles at 1.9 eV observed in both the data and the prediction is likely related to the resonance at 1.75 eV, as indicated by component 4 in Figure 2d.

**Figure** **3** displays the comparison between the experimental and simulated EELS profiles for the edge of the large ${\text{WS}}_{2}$ nanotriangle indicated in the inset. The theoretical prediction, Figure 3b, shows that plasmonic resonances tend to disperse toward lower energy losses as they propagate from the center toward the tip of the triangle, as also observed in the experimental EELS measurements. This behavior, combined with the worse definition of plasmonic modes in dielectrics, leads to slight overlaps between higher‐order plasmon modes at the tips and lower‐order modes at the center of the ${\text{WS}}_{2}$ specimen and a broadening of the associated energy peaks, as indicated by the NMF components 2 and 4 of Figure 2b,d. Inspection of Figure 3 highlights the good agreement between the main qualitative features of the electrodynamical simulations of the EELS edge profiles and the associated experimental measurement is found, confirming their underlying plasmonic nature.

In Figure 3a one observes the third‐order plasmonic resonance mode between 1.7 and 2.0 eV, captured by the NMF component 4, Figure 2d, at ≈1.75 eV and matching with the theoretical prediction in Figure 3b. For higher‐energy losses, both the data and the simulation display increases in the EELS intensity, which can be explained by the fourth‐ and fifth‐order resonance modes as shown in the Figure S6, Supporting Information. In the Section S3.1, Supporting Information the edge dispersions of all triangular ${\text{WS}}_{2}$ nanostructures are compared to their simulated counterparts, see Figure S12, Supporting Information, finding also in this case agreement between data and predictions.

Despite this overall agreement between data and simulations, one should also note that some of the experimental features are not fully reproduced. For instance, the 1.0 eV peak in the experimental data, not captured by the prediction, can be attributed to the first‐order resonance mode of the small ${\text{WS}}_{2}$ triangle leaking to the edge of the large triangle. The same phenomenon is reported for all three edges of the stacked ${\text{WS}}_{2}$ nanotriangles (Figure S11, Supporting Information).

**Figure 4:**
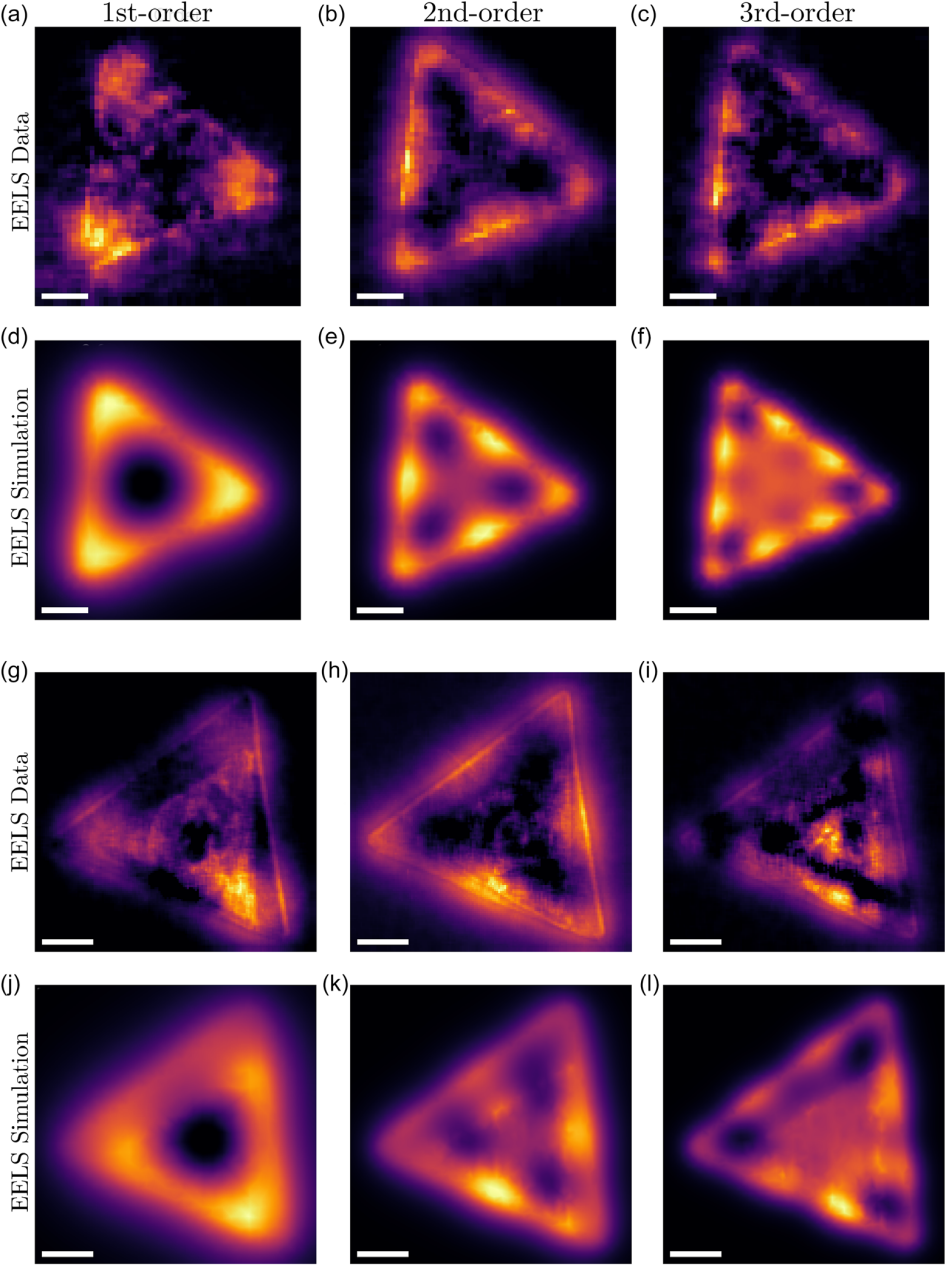
Simulated abundance maps in ${\text{WS}}_{2}$ nanotriangles. Comparison between the experimental and simulated abundance maps in a–f) individual and g–l) stacked ${\text{WS}}_{2}$ nanotriangles. The experimental maps are obtained from the NMF decomposition, while the simulated ones are constructed by filtering the predicted EELS intensity in the energy‐loss window associated to the corresponding endmember of the NMF component. (a–f) For the experimental abundance maps of the 920 nm nanotriangle, we display the first‐, second‐, and third‐order plasmonic resonances whose associated endmembers peak at ΔE=0.91, 1.28, and 1.89 eV respectively, with the corresponding simulated maps filtered to the [0.7,0.9], [1.1,1.3], [1.65,1.85] eV energy windows. (g–l) Same for the stacked nanotriangle, for which the NMF endmembers associated with the first‐, second‐, and third‐order resonances peak at 0.75, 1.22, and 1.74 eV, with the corresponding simulated maps filtered to the [0.7,0.9], [1.1,1.3], and [1.65,1.85] eV intervals. Scale bars correspond to 200 nm.

Next, we compare the abundance maps obtained from the NMF decomposition (e.g., left panels of Figure 2) with the simulated EELS maps for the associated plasmonic resonances. First, in **Figure** **4**a–f we compare the abundance maps of the first, second, and third resonance modes of the standalone triangle with side length of 920 nm, which has a similar morphology to the stacked nanotriangles and hence provides a suitable baseline for the latter. The simulated abundance maps are constructed by filtering the predicted EELS intensity in the energy‐loss window associated with the corresponding endmember of the NMF component. The center and width of this window are determined from a Gaussian fit to the endmembers (e.g., right panels of Figure 2). Good agreement is obtained between the experimental and simulated abundance maps, further validating our interpretation of the EELS data in terms of the localized plasmonic resonances of ${\text{WS}}_{2}$. For the first‐order mode, the bottom‐left corner of the triangle has a higher abundance compared to the other corners, most likely due to the thickness being slightly higher, see Figure S1b, Supporting Information. A similar effect can be seen in the abundance maps of the second‐ and third‐order modes.

Figure 4g–l display the corresponding abundance maps for the stacked ${\text{WS}}_{2}$ nanotriangles, finding also qualitative agreement between data and electrodynamical simulations. Recall from Figure 2 that the NMF components 2, 3, and 4 are found in this specimen to be associated with the first‐, second‐, and third‐order plasmonic resonances. The influence of the thickness on the resonance modes is evident in both experimental and simulation results. In Figure 4i, the abundance map also indicates an increased EELS emission in the center of the structure, a feature absent the simulated maps. Revisiting the thickness map in Figure 1c, this is likely due to the nonuniform thickness of the smaller triangle. Indeed, the slight decrease in the thickness there indicates a hole at the center of the small triangle may contribute to a shift in the EELS response between 1.7 and 2.0 eV, which as a consequence is grouped together with the 1.75 eV resonance in component 4 (Figure 2d).

For completeness, experimental and abundance maps obtained from single‐${\text{WS}}_{2}$ nanotriangles with different sizes are reported in the Supporting Information. Figure S13, Supporting Information displays the maps of the first‐ and second‐order plasmon resonances of the 330 nm nanotriangle and in Figure S14, Supporting Information the first‐, second‐, and third‐order of the 880 nm nanotriangle. Then, in the Figure S15, Supporting Information, we focus on simulating the small triangle with a side length of 680 nm, which is positioned on top of the large triangle. This simulation demonstrates that the localized plasmon resonances mode captured by NMF component 3, Figure 2c, corresponds with those hosted by the small triangle. In Figure S16, Supporting Information, we extend this analysis to confirm that the higher‐order plasmon modes observed in the stacked ${\text{WS}}_{2}$ nanotriangles are indeed a superposition of the fourth‐ and fifth‐order plasmon resonances of the large triangle and the third‐ and fourth‐order plasmon resonances of the small triangle, respectively. These observations align well with the findings from Figure S6, Supporting Information.

Overall, the numerical simulations of the EELS response in ${\text{WS}}_{2}$ nanotriangles with different morphologies are found to be fully consistent with the experimental data, in particular displaying the presence of higher‐order localized plasmonic resonances, therefore providing additional validation for our interpretation of the EELS measurements.

### 2.3 Dispersion Relation of Edge‐Localized Plasmonic Modes

Further scrutinizing the nature of the observed edge‐localized plasmonic ${\text{WS}}_{2}$ modes is possible through the determination of their dispersion relation, namely, the value of the energy $E_{n}$ associated with each of the *n*‐th mode as a function of the corresponding wavenumber $k_{n}$. The indexing for these edge‐localized plasmonic modes is based on a 1D Fabry–Perot model^[^ ^53^, ^54^, ^55^, ^56^, ^57^ ^]^ which has been applied to describe edge modes in metallic triangular nanostructures.^[^ ^58^ ^]^

**Figure 5:**
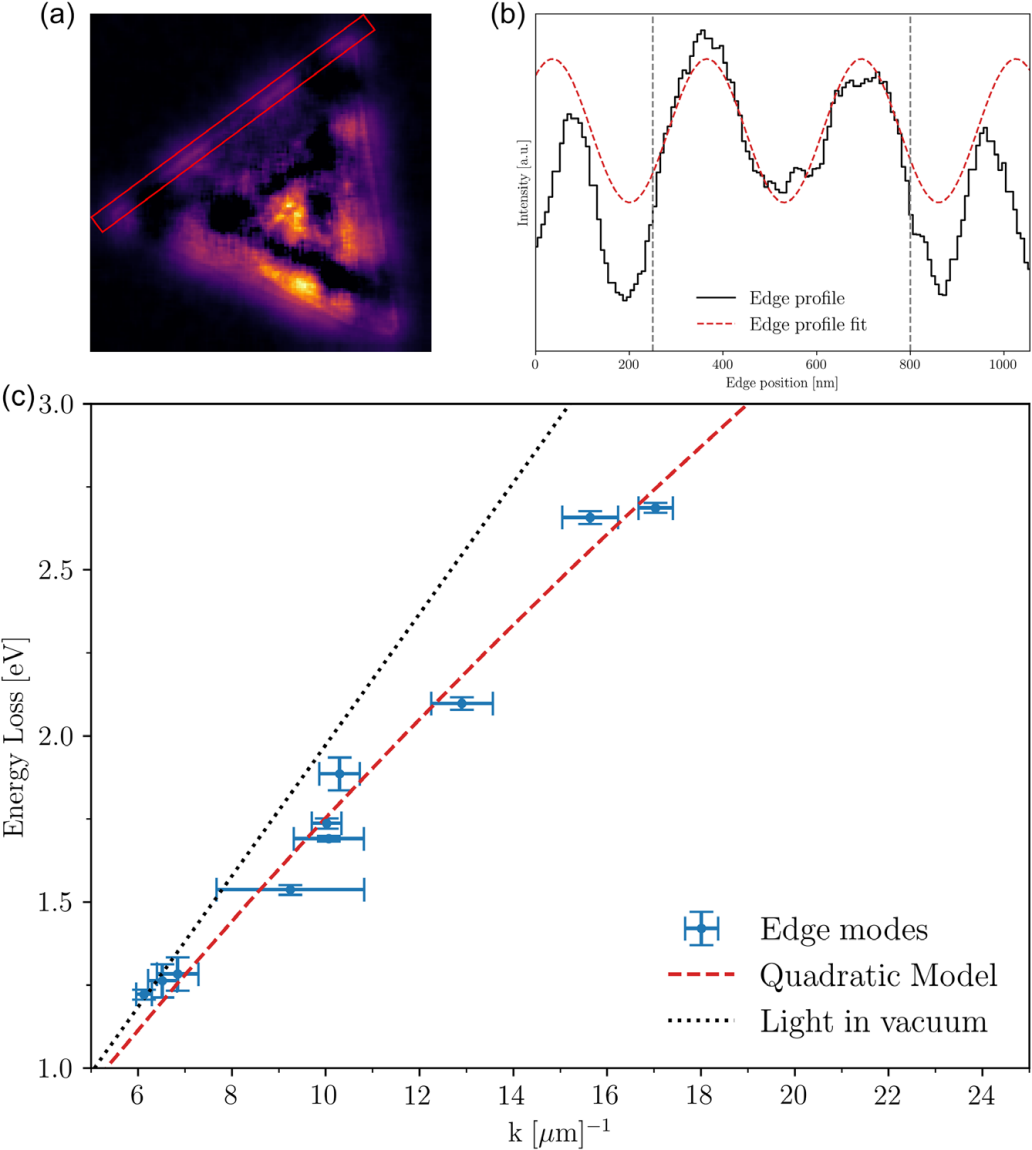
Dispersion relation for the plasmonic edge modes in triangular ${\text{WS}}_{2}$ nanostructures. a,b) Summary of the fitting procedure adopted to extract the phase shift *φ* of the edge profile from the abundance map and entering Equation (1), illustrated in the case of the third‐order resonance mode of the large nanotriangle. The dashed gray lines indicate the fitting ranges. c) The measured values of dispersion relation of the edge‐localized plasmon resonances, with the corresponding uncertainties, are well described by the quadratic polynomial fit, consistently with the expectation for the surface plasmon characteristics. For reference, we also display the dispersion relation of light in vacuum.

In this model, the EELS intensity maps, specifically, the abundance maps of the localized plasmon resonances, arise due to the constructive interference within a 1D Fabry–Perot cavity. This interference occurs when half of the plasmon resonance wavelength *λ* fits within the side length of the triangular nanostructure and can be described by the relation

(1) $$k_{n}L=n\pi -\varphi \;$$

where $k_{n}$ is the wavenumber, *n* is the order of the plasmon resonance, *L* the length of the cavity (nanotriangle side), and *φ* is the phase shift that occurs upon reflection at the boundaries of the nanostructure. This phase shift *φ* can be obtained by fitting the edge profile of the triangle using Equation (S8), Supporting Information, as illustrated in **Figure** **5**a,b.

To illustrate the benefits of adopting NMF decomposition for this application, Figure S18, Supporting Information compares the fourth‐order resonance of the edge profile large triangle edge profile obtained from the NMF abundance map to its counterpart obtained by taking the integrated EELS intensity of the energy window of the resonance. The edge profile obtained from NMF decomposition is much better defined, which is crucial to pin down the underlying nature of the observed resonances.

Figure 5c displays the experimentally measured values of $E_{n}$, the peak energy of the *n*‐th plasmonic resonance obtained from the NMF endmembers, as a function of the associated wavenumbers $k_{n}$ computed from Equation (1), together with the associated uncertainties. The measured values for the dispersion relation $E_{n}=f(k_{n})$ are fitted to a quadratic model of the form $E_{n}=Ak_{n}^{2}+Bk_{n}+C$, which is the expected behavior given the surface plasmon characteristics,^[^ ^55^, ^59^, ^60^, ^61^ ^]^ finding good agreement. This polynomial fit is compared to a linear fit in Figure S17, Supporting Information, showing that the latter is insufficient to describe the experimental values. For reference, Figure 5c also displays the dispersion relation associated with light propagating in vacuum.

The confirmation presented in Figure 5c that the dispersion relations of the observed edge‐localized periodic modes in our ${\text{WS}}_{2}$ nanostructures is well described by a quadratic model provides yet another piece of evidence demonstrating the plasmonic nature of the observed resonances from the EELS data.

## 3 Summary and Outlook

In this study we have fingerprinted edge‐localized plasmonic resonances arising in single and stacked ${\text{WS}}_{2}$ nanotriangles from a synergetic combination of spatially‐resolved EELS and NMF techniques. Our approach successfully resolves edge‐localized plasmonic modes up to the fourth‐order resonance in single nanotriangles and up to the fifth‐order resonance in the stacked nanotriangles. In particular, we are able to disentangle the main features of the localized plasmonic resonances appearing in the smaller triangle for the stacked configuration, which would have been otherwise obscured by the bulk signal of the larger bottom triangle if one had relied on more traditional methods.

To validate the interpretation of our EELS‐based analysis, we have compared the results with dedicated electrodynamical simulations of the EELS edge profiles based on the pygdm package, finding good agreement in terms of both the abundance maps of the localized plasmonic resonances and on the values of the associated energies for each resonance mode. Furthermore, the experimental determination of the dispersion relation of the edge‐localized plasmonic modes obtained from the endmembers of the NMF decomposition is found to be well described by a quadratic model (but not by a linear one), consistently with the expected behavior for surface plasmon characteristics.

All in all, our findings highlight the potential of boosting spatially resolved EELS with sophisticated data processing algorithms, in this case NMF supplemented with machine learning for the model‐independent subtraction of the ZLP. This potential is most effective for analyses of nanospecimens with nontrivial morphologies, such as the overlapping ${\text{WS}}_{2}$ nanotriangles considered in this work, where conventional methods do not suffice to identify all the relevant features present in the data. More toward the future, the methodology presented in this work opens new avenues in exploiting the capabilities of localizing plasmonic resonances with well‐defined spectral characteristics to harness light–matter interactions in TMD materials, improving prospects for their applications to optoelectronic and nanophotonic devices, such as photodetectors with high spatial resolution. Exploiting the edge‐localized plasmonic resonances for enhanced light absorption and local electric fields is another promising avenue,^[^ ^21^ ^]^ as well as their use as nanoscale light sources,^[^ ^62^ ^]^ and their application to frequency conversion and ultrafast laser technologies in plasmon‐enhanced nonlinear optics.^[^ ^63^ ^]^

## 4 Experimental Section

4.1

4.1.1

##### Fabrication of ${\mathrm{WS}}_{2}$ *Nanotriangles*

The twisted ${\text{WS}}_{2}$ specimens were grown directly on a silicon TEM grid with nine windows, each spanned by a 5 nm‐thick ${\text{Si}}_{3}N_{4}$ membrane. Tungsten trioxide (${\text{WO}}_{3}$) powder, which acts as a seeding material, was deposited on this substrate by dispersing 50 mg of ${\text{WO}}_{3}$ in 1 mL of isopropanol. A few drops of this solutions were then deposited onto the substrate using a pipette. After the sample was left to dry, ${\text{WO}}_{3}$ was successfully spread over the substrate. Following this preparation, the ${\text{WO}}_{3}$‐coated substrate was placed in the central heating zone of a gradient tube furnace from Carbolite Gero. A crucible containing sulfur powder was positioned in a separate heating zone upstream. The central heating zone was heated to a temperature of 750 °C, followed by heating the zone containing the sulfur powder to 220 °C. The system was maintained at these temperatures for 1 h under a consistent argon flow of 150 sccm. Before this process, the system was flushed using an argon flow of 500 sccm for 30 min. Once the reaction time passed, the furnace was cooled down naturally.

##### STEM–EELS Analyses

The STEM–EELS measurements were performed on an ARM200F Mono‐JEOL microscope. The microscope was operated at 200 kV with the monochromator ON and a slit of 1.3 μm inserted for the stacked nanotriangles and a slit of 2.0 μm for the single nanotriangles. A Gatan GIF Quantum ERS system (model 966) was used for the EELS acquisition. The convergence and collection semiangles were 19.96 and 14.5 mrad respectively. EELS of the stacked nanotriangles were acquired with an entrance aperture diameter of 5 mm, energy dispersion of 0.015 eV/channel, and a pixel time of 0.5 s, resulting in a ZLP full width at half maximum (FWHM) of 0.12 eV. For the single nanotriangles, the EELS were acquired with the same entrance aperture diameter of 5 mm, but with an energy dispersion of 0.05 eV/channel, and a pixel time of 1.0 s, yielding ZLP FWHM of 0.25 eV.

## Conflict of Interest

The authors declare no conflict of interest.

## Author Contributions

**Abel Brokkelkamp**: formal analysis (lead); investigation (equal); methodology (lead); visualization (lead); writing—original draft (lead); writing—review and editing (equal). **Sabrya E. van Heijst**: writing—original draft (supporting); writing—review and editing (supporting). **Sonia Conesa‐Boj**: funding acquisition (lead); supervision (lead); writing—original draft (equal); writing—review and editing (equal).

## Supporting information

Supplementary Material

## Data Availability

The data that support the findings of this study are available from the corresponding author upon reasonable request.
