# Supplementary material for: Edge‐Localized Plasmonic Resonances in WS2 Nanostructures from Electron Energy‐Loss Spectroscopy
Source: Small Sci. 2025 Feb 13;5(5):2400558. doi: 10.1002/smsc.202400558 (PMC12087782; doi:10.1002/smsc.202400558)
Supplement: Supplementary file 1 — Supplementary Material [file SMSC-5-2400558-s001.pdf]

Edge-Localised Plasmonic Resonances in WS_2 Nanostructures from Electron Energy-Loss Spectroscopy: Supplementary Information

Abel Brokkelkamp[1], Sabrya E. van Heijst[1], Sonia Conesa-Boj[1,*]

[1]Kavli Institute of Nanoscience, Delft University of Technology, 2628 CJ, Delft, The Netherlands

*Corresponding author. Email: s.conesaboj@tudelft.nl

Contents

S1 Structural Characterization of WS_2 Nanostructures **2**
S1.1 WS_2 Nanostructure Thickness Mapping 2
S1.2 Morphology and Thickness Mapping of Additional WS_2 Nanostructures 2

S2 Spatially-Resolved EELS Data Processing **4**
S2.1 ZLP Subtraction with Deep Learning Methods 4
S2.2 Decomposition of EELS data using NMF 7
S2.3 NMF decomposition of WS_2 nanotriangles 8
S2.4 Validation of the NMF Abundance Maps from EELS Point Spectra 9

S3 EELS-GDM Electrodynamical Simulations **16**
S3.1 Edge Dispersions of Nanotriangles 16
S3.2 EELS Probability Maps 19
S3.3 Potential Limitations of the Simulation Model 20

S4 Extracting Edge Modes in WS_2 nanotriangles from NMF **24**
S4.1 Advantages of NMF Over Conventional Intensity Mapping Methods 25

S1 Structural Characterization of WS_2 Nanostructures

Here we present the morphological and structural analysis of WS_2 nanotriangles with different side lengths. This characterization, using scanning transmission electron microscopy-high angle annular dark-field (STEM-HAADF) imaging and thickness mapping, provides essential insights into how size and thickness variations impact the plasmonic properties of the nanotriangles. This analyses also allow comparisons between single and stacked triangular structures, revealing the potential for larger or thicker structures to support higher-order plasmonic modes.

S1.1 WS_2 Nanostructure Thickness Mapping

Fig. 1c in the main manuscript shows the thickness map of the specimen, calculated using the log-ratio method and mass density of the material to determine the mean free path of the electrons [1]. After subtracting the thickness of the Si_3N_4 membrane, the large triangle has an average thickness of approximately 30 nm, with side lenghts measuring around 980 nm. The small triangle has an average thickness of approximately 35 nm, after accounting for the thickness of the Si_3N_4 membrane and the large triangle. However, it exhibits greater variation in thickness, ranging from about 30 nm to 50 nm, with side lenghts measuring around 680 nm. In both triangles, the corners are slightly truncated, and the edges are slightly tapered.

S1.2 Morphology and Thickness Mapping of Additional WS_2 Nanostructures

Figures S1 trough S3 display the morphology of WS_2 nanotriangles with side lengths of 330 nm, 880 nm, and 920 nm, using STEM-HAADF and corresponding thickness maps. These morphological details provide a basis for comparing the influence of size and thickness on plasmonic behaviour, specifically showing how larger or thicker nanotriangles may support higher-order plasmon modes.

The WS_2 nanotriangle presented in Fig. S1 has side lengths of approximately 920 nm and average thickness of about 70 nm, with the top right side being slightly thinner than the bottom left. This thickness closely resembles that of the stacked WS_2 nanotriangles, making it a suitable morphological comparison in the main text.

The next WS_2 nanotriangle, shown in Fig. S2, has side lengths of approximately 330 nm and and average thickness of about 85 nm, with the top part being the thinnest and the bottom part showing the greatest thickness. The final WS_2 nanotriangle presented in Fig. S3, has side lengths of approximately 880 nm. Notable defects include a more truncated tip on the left side and the gap on the right, with spikes near the gap location. This nanotriangle is significantly thicker than the others, averaging around 225 nm, with the top thinner than the bottom. The gap and proximity of the beam to the nanostructure lead to an increase in the overall spectral intensity, slightly distorting the thickness measurement.

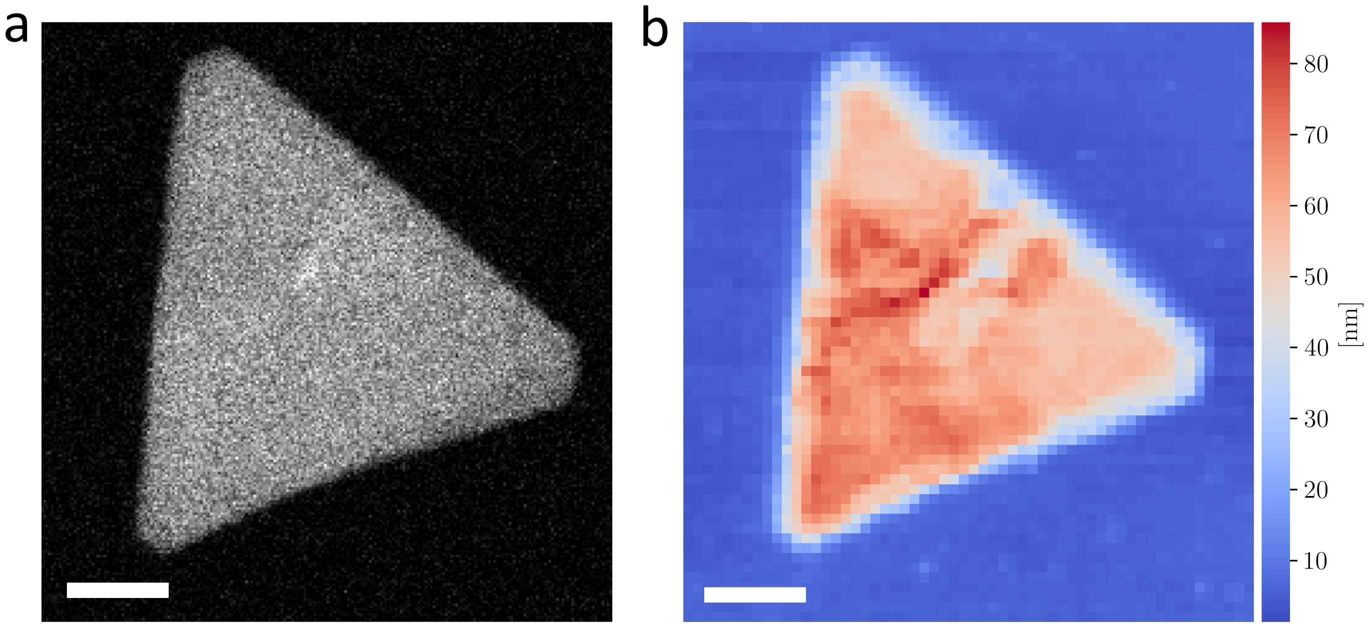

Figure S1: Morphology of the WS_2 triangular nanotriangle with a side length of approximately 920 **nm. (a)** STEM-HAADF image illustrating the structural features. **(b)** Thickness map with an average thickness of around 70 nm, showing thickness variations from the top right to the bottom left. Scale bars are 200 nm.

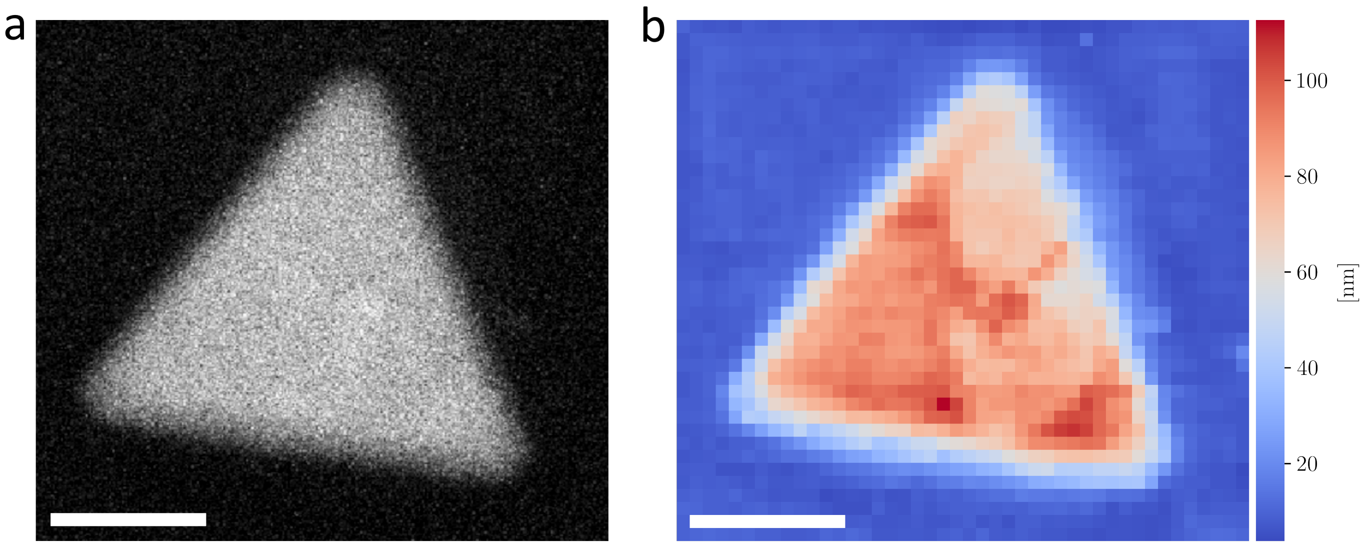

Figure S2: Morphology of a WS_2 nanotriangle with a side length of approximately 330 **nm. (a)** STEM-HAADF image showing the overall structure. **(b)** Thickness map with an average thickness of about 85 nm. Scale bars represent 100 nm.

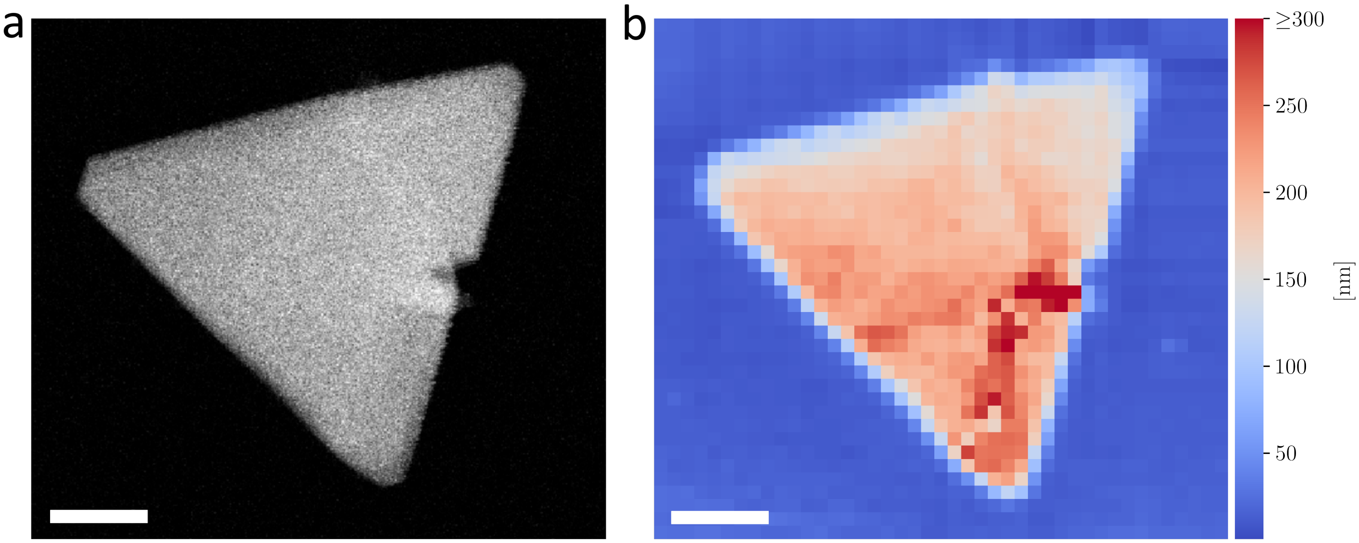

Figure S3: Morphology of a WS_2 nanotriangle with a side length of approximately 880 **nm. (a)** STEM-HAADF image showing structural details, including notable defects. **(b)** Thickness map with and average thickness of about 225 nm. Notable defects include a truncated tip on the left and a gap on the right side, causing intensity distortions near the gap. Scale bars represent 200 nm.

S2 Spatially-Resolved EELS Data Processing

In this section, we outline the processing steps for electron energy loss spectroscopy (EELS) data obtained from WS_2 nanotriangles. The methods include Zero Loss Peak (ZLP) subtraction using a deep-learning approach, followed by decomposition of the EELS data using non-negative matrix factorization (NMF). These steps are essential for isolating meaningful spectral features and improving the accuracy of plasmonic and electronic property analysis across different energy windows.

S2.1 ZLP Subtraction with Deep Learning Methods

The ZLP, which often overlaps with the specimen signal, presents a challenge for separation using conventional methods, particularly in cases of significant overlap, as shown in Fig. S4b. To address this, we have developed a deep-learning approach available in our open-source Python package, EELSFITTER, which has proven effective in prior studies. Full detail of the methodology are described in recent publications of our group [2, 3]; here, we provide a brief summary.

The ZLP is parameterized by two input variables: the energy loss E, where the ZLP intensity is measured, and the logarithm of an intensity value, $\ln(N_{peak})$, which correlates with the ZLP intensity and serves as a proxy for sample thickness. In thicker samples, the ZLP (and thus N_{peak}) is smaller, and vice versa.

The output of the network is the logarithm of the ZLP intensity, $\ln\left(I_{\rm ZLP}^{(NN)}\left(E, \ln\left(N_{\rm peak}\right)\right)\right)$, based

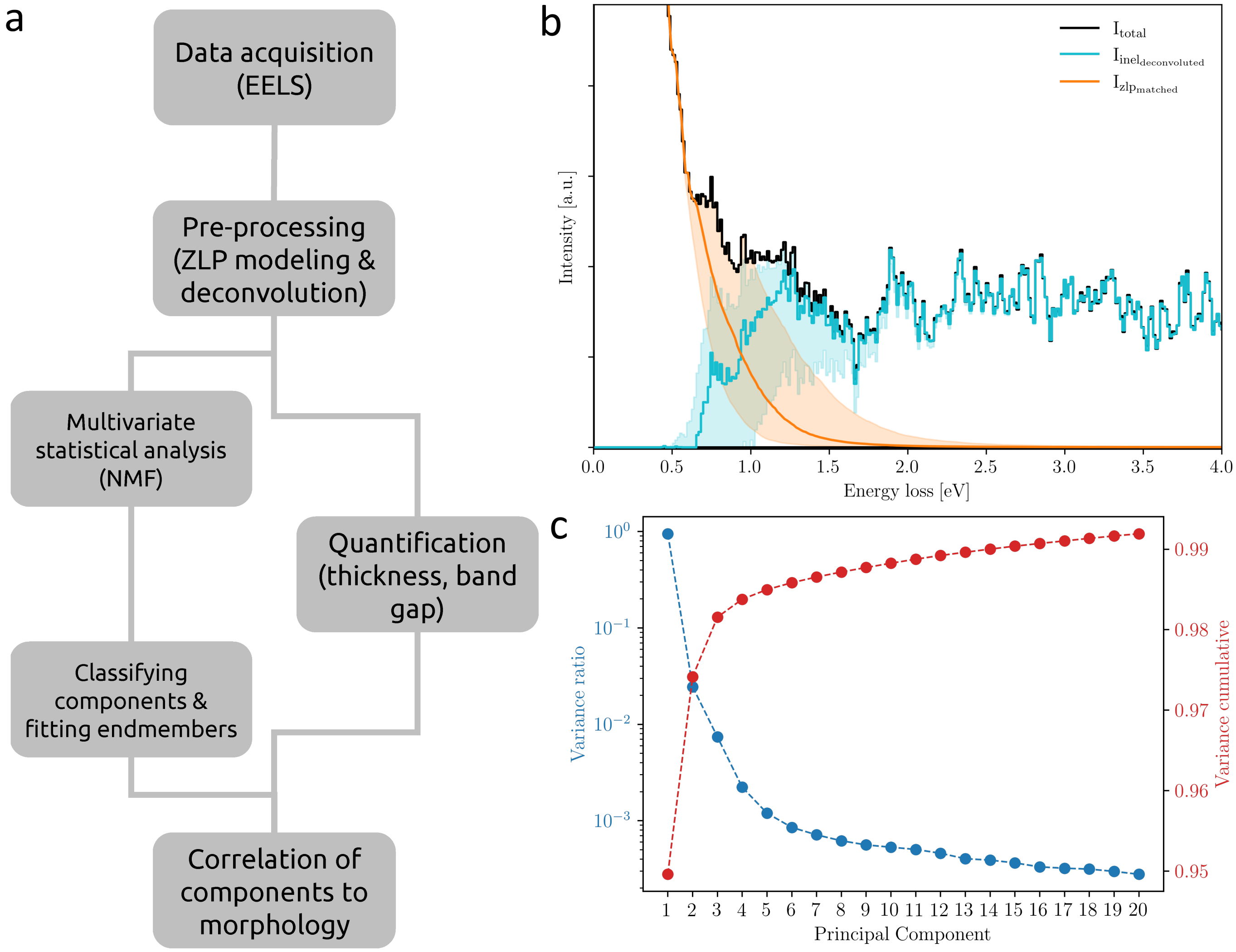

Figure S4: Data Processing of EELS Spectral Images. (a) Schematic of the EELS data processing steps. **(b)** Deconvolution of the ZLP from a representative EEL spectrum, with the orange shaded area representing the 90% confidence level (CL) interval obtained from the trained ZLP models. **(c)** Scree plot from principal component analysis (PCA) applied to the ZLP-subtracted EELS data within the 0.5 to 2.5 eV energy window. From the variance ratio and the cumulative variance, we determine the first four components to be most statistically significant (adding up to 98.5% of the total variance). These first four components are discussed in Fig. 2 in the main text.

on these input variables. Taking logarithmic values aligns the magnitude of input and output values, which benefits the neural network architecture by stabilizing sigmoid activations used in each layer. The final layer employs ReLU activation to ensure a positive output.

A key feature of this approach is its capacity to quantify uncertainties in ZLP modelling. Furthermore, it does not impose assumptions about ZLP shape, instead adapting flexibility to variations. Prior to training, K-means clustering groups the spectral image into K clusters based on local thickness, so ZLPs in each cluster are expected to follow similar distributions. This setup enables probability sampling of the EEL spectra allowing a large number of neural networks to be trained in parallel trough Monte Carlo sampling. In this Monte Carlo Replica method, neural networks are trained on spectra randomly sampled from each cluster. The combined network outputs yield a probability density representation of the ZLP distributions across intensity values. As a result, statistical quantities such as the median prediction and confidence level intervals can be computed. Each replica is represented as,

$$\boldsymbol{I}^{(m)} = \left\{ I_{\rm EELS}^{(i_{m,1},j_{m,1})}(E), I_{\rm EELS}^{(i_{m,2},j_{m,2})}(E), \ldots, I_{\rm EELS}^{(i_{m,K},j_{m,K})}(E) \right\}, \quad m = 1, \ldots, N_{\rm rep}, \tag{1}$$

where $I_{\rm EELS}$ is the recorded intensity, and $(i_{m,k}, j_{m,k})$ identifies a spectrum from cluster k ($k = 1, \ldots, K$) assigned to replica m.

The neural network is trained by minimising a cost function tailored for each replica as follows:

$$\begin{aligned} C_{\rm ZLP}^{(m)} = & \frac{1}{n_{E_{\rm I}}} \sum_{k=1}^{K} \sum_{\ell_k=1}^{n_{E_{\rm I}}^{(k)}} \frac{\left[I_{\rm EELS}^{\left(i_{m,k},j_{m,k}\right)}\left(E_{\ell_k}\right) - I_{\rm ZLP}^{(\rm NN)(m)}\left(E_{\ell_k}, \ln\left(N_{\rm peak}^{\left(i_{m,k},j_{m,k}\right)}\right)\right)\right]^2}{\sigma_k^2\left(E_{\ell_k}\right)} \\ & + \lambda \sum_{k=1}^{K} \sum_{r_k=1}^{n_{E_{\rm II}}^{(k)}} \mathrm{ReLU}\left(\frac{\mathrm{d}I_{\rm ZLP}^{(\rm NN)(m)}\left(E_{r_k}, \ln\left(N_{\rm peak}^{\left(i_{m,k},j_{m,k}\right)}\right)\right)}{\mathrm{d}E} \right), \\ & E_{\ell_k} \leq E_{{\rm I},k}, \quad E_{{\rm I},k} \leq \Delta E_{r_k} \leq E_{{\rm II},k}, \end{aligned} \tag{2}$$

where $I_{\rm ZLP}^{(\rm NN)(m)}$ is the ZLP intensity predicted by the neural network for replica m. Here, $n_{E_{\rm i}}^{(k)}$ represents the number of energy bins in region i of the spectrum from cluster k, $n_{E_{\rm I}}$ is the total across clusters, $\sigma_k\left(E_{\ell_k}\right)$ is the variance within cluster k at the energy loss E_{ℓ_k}, and λ balances the two terms in the cost function.

The spectra used for training are divided into three regions I, II and III. In region I the intensity is dominated by the contribution from the ZLP. In region II the intensity of the ZLP tail and inelastic scattering contribution of the specimen are comparable. Finally, in region III the contribution of the ZLP is approximately zero. These regions are delineated by hyperparameters, $E_{{\rm I},k}$ and $E_{{\rm II},k}$, which are automatically determined from the data using the kneedle algorithm. This algorithm identifies the point of highest curvature in a concave or convex curve, which we use to separate ZLP and inelastic scattering contributions. To define the kneedle point between the ZLP and the

specimen's scattering contribution, we construct a concave curvature from Full Width at Half Maximum (FWHM) and the first local minimum between these contributions. $E_{\mathrm{I},k}$ is set at the kneedle location, adjusted slightly by a scaling factor. $E_{\mathrm{II},k}$ is determined by fitting a logarithmic function through the kneedle point and the local minimum, using the intersection at a single count as the threshold (representing the minimal possible ZLP contribution).

S2.2 Decomposition of EELS data using NMF

Non-negative matrix factorization (NMF) is a multivariate analysis (MVA) algorithm often referred to as blind-source separation (BSS). Another example of such family of techniques is principal component analysis (PCA), which has already seen widespread use in EELS applications.

The spectral image can be expressed as a three-dimensional matrix, $D(x, y, \Delta E)$, where (x, y) denotes the two-dimensional probe position from which spectral data is collected, and ΔE denotes the energy channels of the spectrometer. To facilitate analysis, the spectral image can be reshape into a two-dimensional matrix $\mathbf{X}(n_{xy}, n_{ch})$, where $n_{xy} = n_x \times n_y$ is the total number of probe positions (pixels), and n_{ch} (= ΔE) is the number of energy channels.

The information within an EEL spectrum is assumed to be a linear combination of basis spectra linked to underlying features and excitations. If there are k basis spectra, with k significantly smaller than the matrix dimensions $n_{xy} \times n_{ch} \gg n_{xy} \times k + n_{ch} \times k$, we can store the basis spectra in matrix $\mathbf{W}$ $n_{ch} \times k$ and the concentration coefficients in matrix $\mathbf{H}$ $n_{xy} \times k$. The original matrix $\mathbf{X}$ can then be approximated as:

$$\mathbf{X} \approx \mathbf{W}\mathbf{H}^T \tag{3}$$

where the superscript T denotes the transpose. In MVA terminology, $\mathbf{H}$ is called the loading matrix, with each column vector (or loading) representing an "endmember" in the main text. Each endmember corresponds to a basis spectrum, ideally capturing a pure signal of the feature it represents. $\mathbf{W}$, known as the score matrix, contains scores in each column vector, representing the weight of the endmember at each location, referred to as the "abundance" in the main text.

As previously mentioned, PCA is the most widely known type of multivariate analysis (MVA) algorithm, with a common application being noise reduction. One important property of PCA is that its principal components are orthogonal and uncorrelated. This means that the first component captures the greatest variance in the data, and each subsequent component maximizes the remaining variance while maintaining orthogonality to all previous components. This structure allows for the creation of a scree plot, as seen in Fig. S4c, which makes it possible to determine the number of statistically relevant components.

EELS data consists of spectroscopic counts that are inherently positive, as negative counts are unphysical. However, when PCA is applied to EELS data, the first component typically represents an averaged base spectrum, but subsequent components, constrained by orthogonality, may contain negative values in their endmembers and abundances. This characteristic often complicates the physical interpretation of the components.

NMF differs from PCA by solving Eq. (3) with the constraint that **X**, **W**, and **H** are all non-negative. The absence of negative values in NMF components provides a more interpretable solution for spectroscopic data. However, a consequence of this non-negativity constraint is that the components are no longer orthogonal. Matrix **W** and matrix **H** are thus numerically approximated by minimizing the following loss function:

$$\|\mathbf{X} - \mathbf{W}\mathbf{H}^T\|_F \tag{4}$$

Here F denotes as the Frobenius norm. Several approaches have been developed to use this form as the basis for minimizing and obtaining solution for **W** and **H**. Although NMF and PCA differ in their component-determination methods, they share the goal of image decomposition, allowing us to use a PCA-derived scree plot as a rough guide for the number of components needed for NMF.

For EELS data, proper normalisation is essential before performing NMF or PCA. In this case, normalisation involves dividing each pixel's spectrum by its ZLP intensity. This step is crucial because the probability of electron interactions is independent of material thickness, but the counts reaching the detector may vary with thickness. For instance, a plasmonic feature may yield higher counts along or near an edge compared to the bulk, due to thickness effects.

S2.3 NMF decomposition of WS_2 nanotriangles

In Fig. 2 of the main manuscript, we examine energy windows between 0.5 to 2.5 eV to decompose the spectral image. Narrowing the energy window can benefit the decomposition process by enhancing feature separation. The exception to this rule is the energy window of the smallest triangle (0.5 to 4.0 eV, as there are a lower amount of resonance modes to be separated (see Fig. S12(c,d).

In Fig. S5 we present the same as main text Fig. 2, but with an additional fifth component. From the scree plot in Fig. S4(c) this component is arguably on the side of statistical insignificance, but as the endmember and abundance map do show a distinct feature (that being the second-order mode of the smaller nanotriangle on top), we present it here. The EELS simulation in Fig. S15 of the small triangle do indicate this being a genuine feature otherwise not observed in the experimental data.

In Fig. S6, data in the energy window between 2 and 4 eV is decomposed, with three components best describing this range. Components 2 and 3 represent higher-order plasmon modes in both the larger and smaller triangle. Component 2 displays the fourth-order mode in the large triangle and the third-order mode in the small triangle, as shown in the abundance map, the latter being less well defined here. Due to the overlapping energy values of the plasmon modes of the small and large triangle, we do not observe a difference in the endmember, but do see both modes in the abundance map. The higher order resonances modes are less well defined and spectrally more spread out. The dominating 2.1 eV peak is associated with the fourth-order plasmon resonances of the large triangle and a bump at 2.3 eV to the third-order plasmon mode in the small triangle, consistent with the EELS-GDM simulations in Figures S11 and S12. Similarly, Component 3 represents the fifth-order plasmon mode in the large triangle and the fourth-order mode in the small triangle,

both near 2.75 eV as indicated by the EELS-GDM simulations. Component 4 is most likely related to the bulk WS_2 crystal, specifically the surface of the large triangle. The rest of this section presents NMF decomposition of additional WS_2 nanotriangles.

We used the NMF implementation in SCIKIT-LEARN version 1.5.2 for the results presented in this work. Most parameters in the NMF algorithm were kept at their default values, with two exceptions. First, the `solver` parameter was set to 'multiplicative update,' as it provided significantly better and faster results compared to the 'coordinate descent' option. Second, the `maximum_iterations` parameter was set to 10^5, which we confirmed is sufficiently large to ensure convergence for all cases relevant to our analysis.

S2.4 Validation of the NMF Abundance Maps from EELS Point Spectra

To further confirm the presence of the features identified by the endmembers of the NMF decomposition and their abundance, we compare the NMF abundance maps to EELS point spectra selected in areas of the specimen corresponding to high and low abundance in the NMF maps. In Fig. S10 we compare the abundance maps of components 2 to 4 as provided in the main text in Fig. 2, where the locations of the plasmon modes associated to the relevant endmembers are indicated by the grey dotted vertical lines. In all three cases considered, we clearly observe the presence of the expected features in the EELS point spectra taken from areas of high abundance, as well as the absence or significant reduction of the same features in the point spectra taken from areas of low abundance.

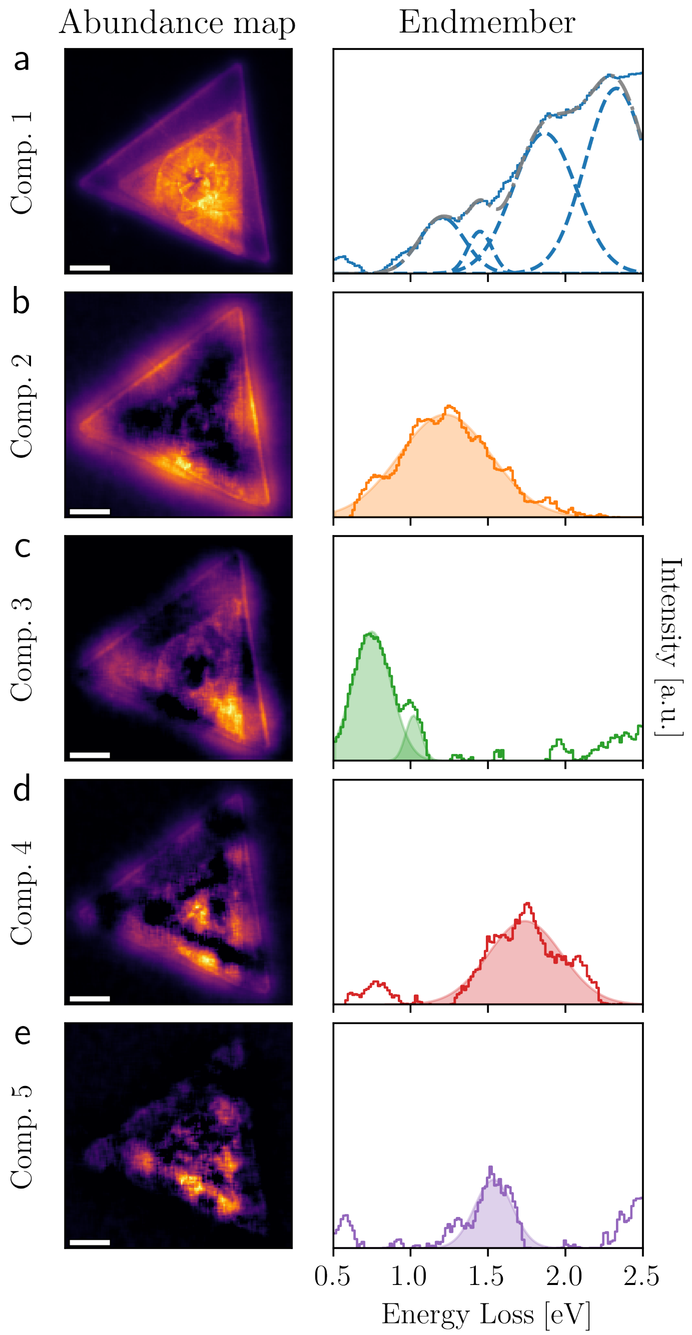

Figure S5: NMF decomposition of the stacked WS_2 nanotriangles. (a-d) The abundance maps (left) and endmembers (right panel) of the first four components of the NMF decomposition from the stacked WS_2 nanotriangles, same as Fig. 2 now with the results of Gaussian fits to the endmembers (right panel). The dashed Gaussians in component 1 represent crystal-structure specific features such as excitons. The shaded Gaussians in components 2-4 indicate the characteristics energies of the plasmonic resonance modes of different order. **(e)** Same as (a-d) now for the fifth component of the NMF decomposition, which can be associated to the 2nd-order resonance mode of the smaller triangle as shown in Fig. S15. All vertical axes are uniformly scaled. Scale bars represent 200 nm.

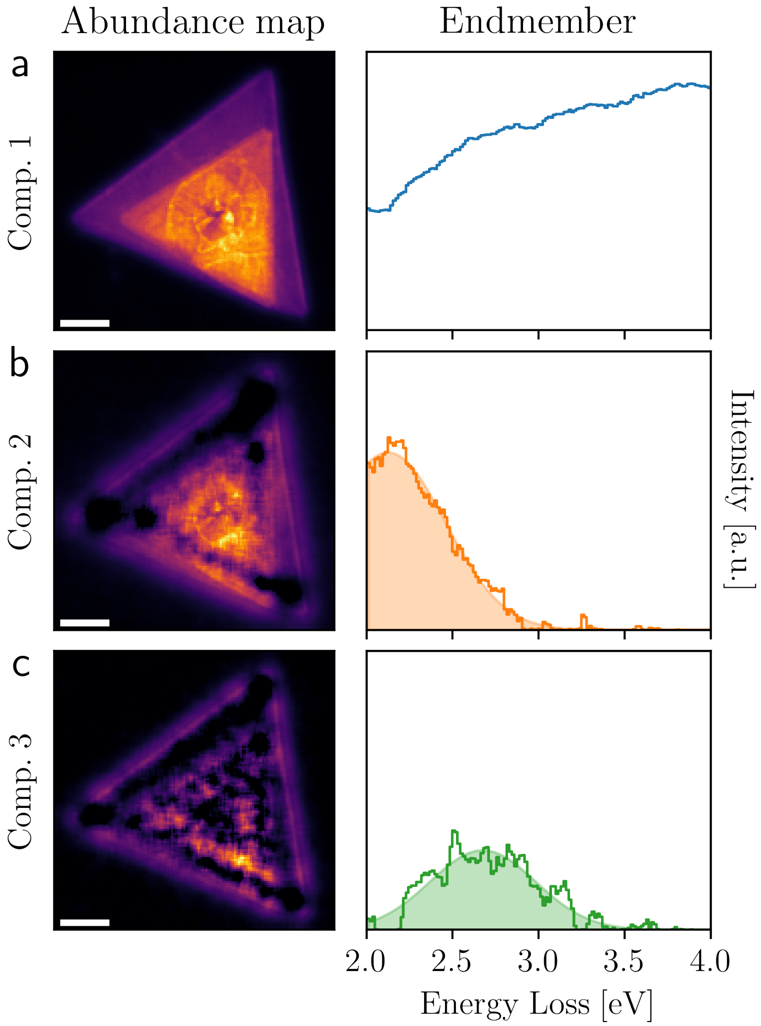

Figure S6: NMF components from energy window 2.0 **to** 4.0 **eV. (a)** Component 1 represents the bulk WS_2 spectrum. **(b)** Component 2 represents the 4th-order resonance mode of the large triangle and possibly the 3rd-order resonance mode of the small triangle. **(c)** Component 3 represents the 5th-order resonance mode of the large triangle and possibly the the 4th-order resonance mode of the small triangle. Scale bars represent 200 nm

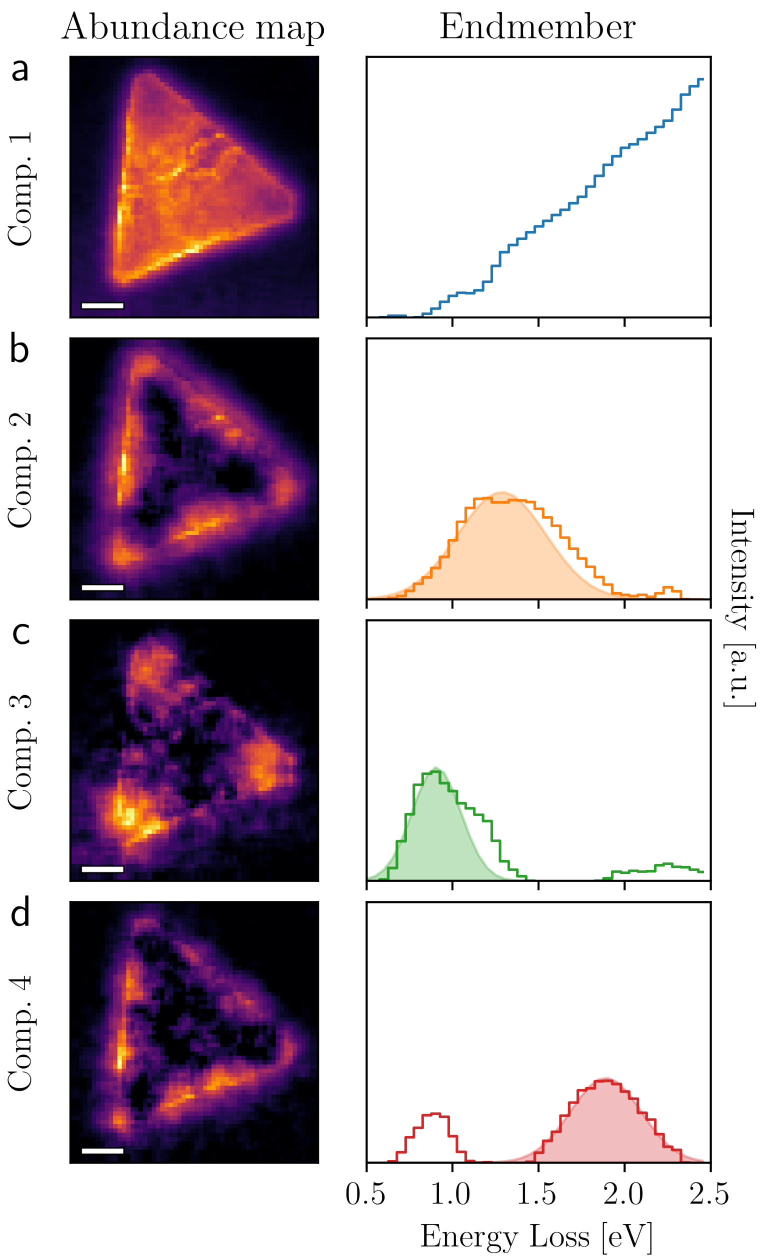

Figure S7: NMF components of WS_2 nanotriangle with 920 **nm side lengths, energy window** 0.5 **to** 2.5 **eV. (a)** Component 1 predominantly represents the bulk WS_2 spectrum. **(b)** Component 2 represents the 2nd-order resonance mode. **(c)** Component 3 represents the 1st-order resonance mode. **(d)** Component 4 represents the 3rd-order resonance mode. Scale bars represent 200 nm

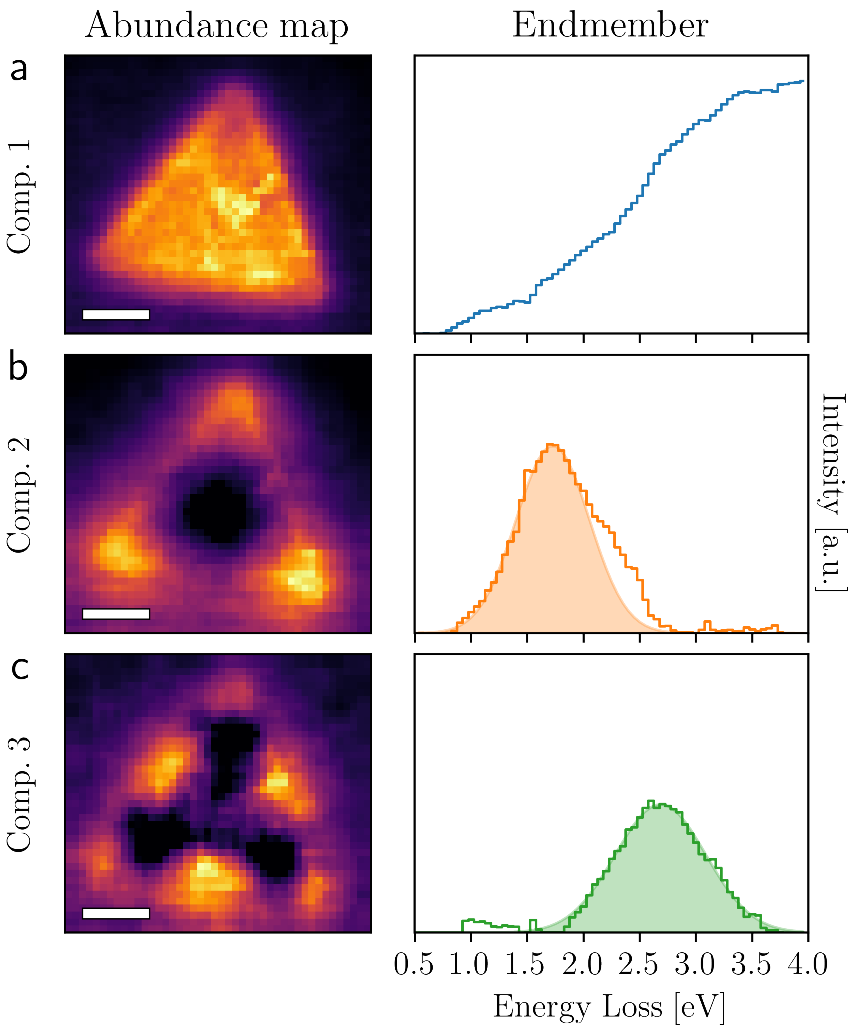

Figure S8: NMF components of WS_2 nanotriangle with 330 **nm side lengths, energy window** 0.5 **to** 4.0 **eV. (a)** Component 1 predominantly represents the bulk WS_2 spectrum. **(b)** Component 2 represents the 1st-order resonance mode. **(c)** Component 3 represents the 2nd-order resonance mode. Scale bars represent 100 nm

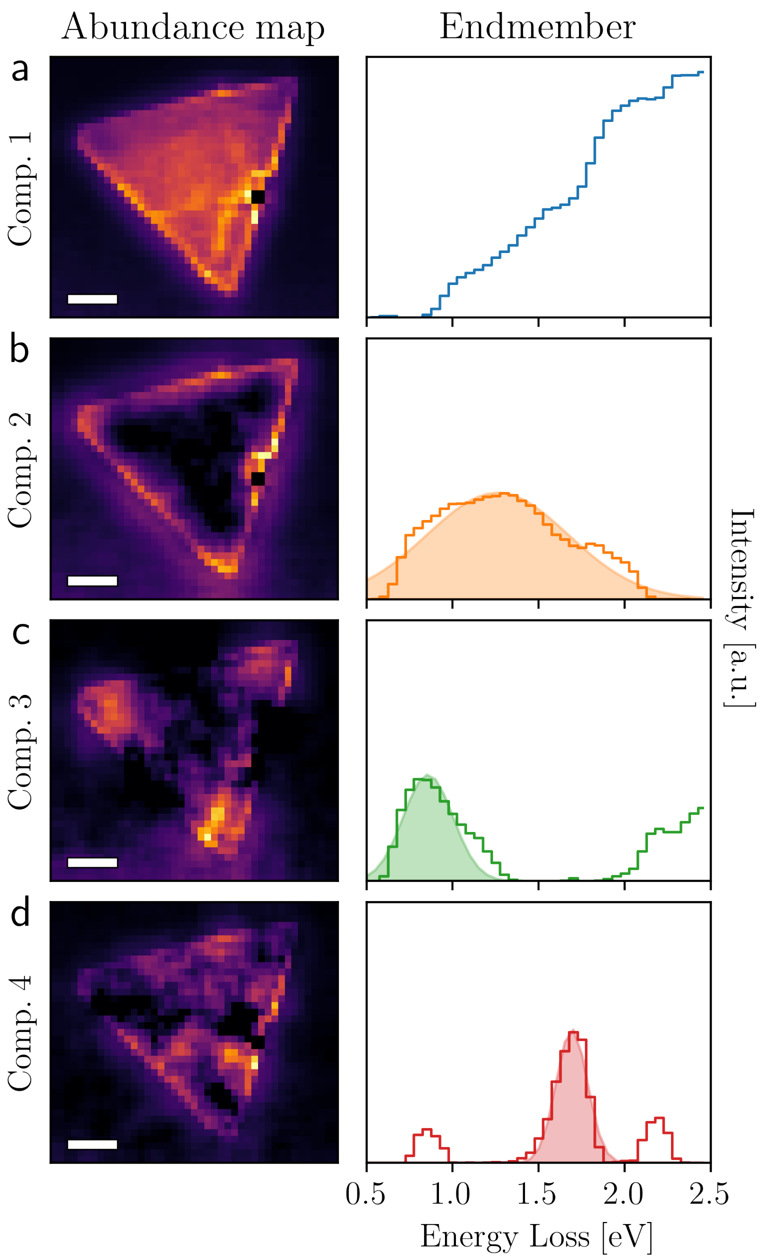

Figure S9: NMF components of WS_2 nanotriangle with 880 **nm side lengths, energy window** 0.5 **to** 2.5 **eV. (a)** Component 1 predominantly represents the bulk WS_2 spectrum. **(b)** Component 2 represents the 2nd-order resonance mode. **(c)** Component 3 represents the 1st-order resonance mode. **(d)** Component 4 represents the 3rd-order resonance mode. Scale bars represent 200 nm

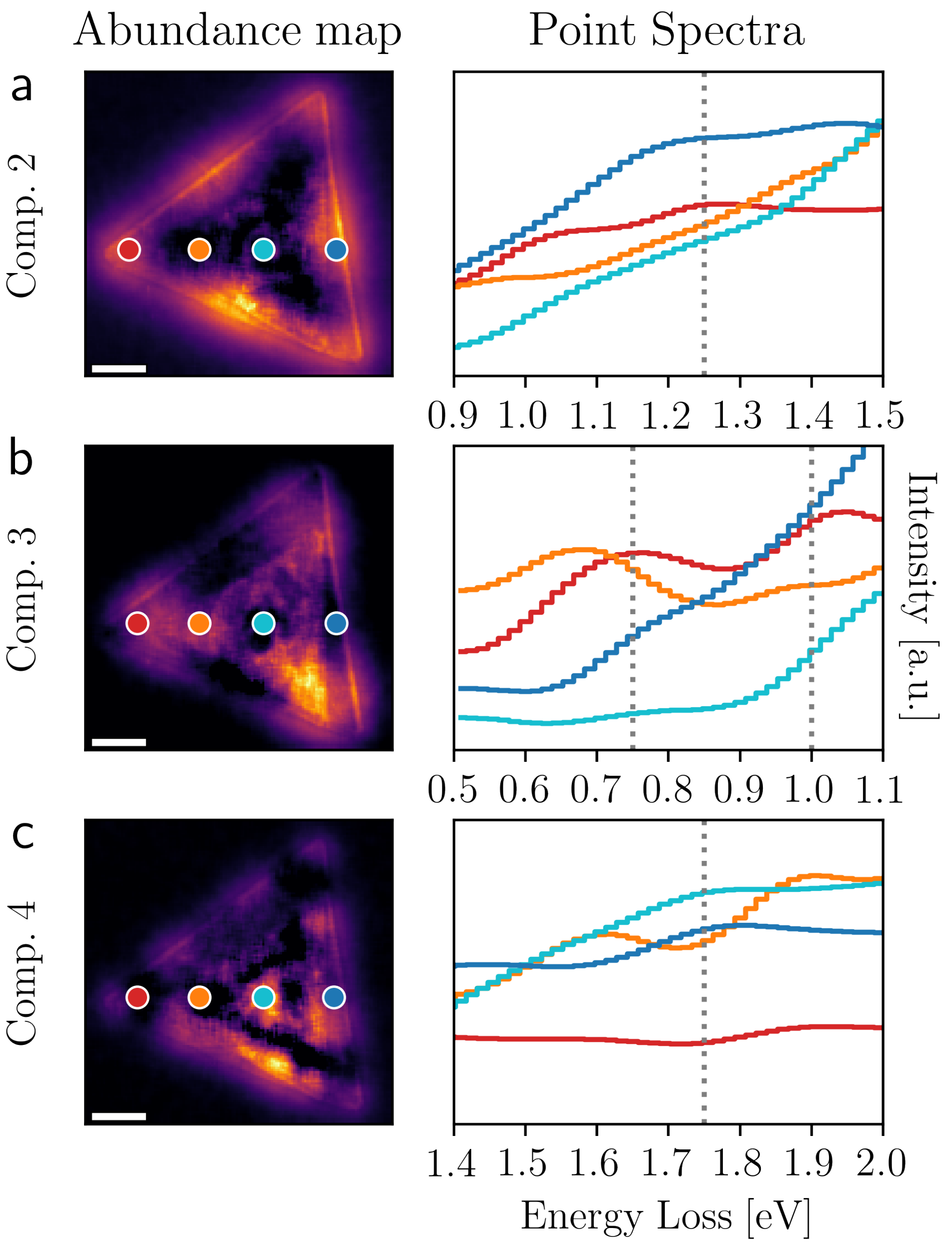

Figure S10: Additional validation of the NMF abundances maps with EELS point spectra. The NMF abundance maps of components #2, #3, and #4 extracted from Fig. 2 are compared to EELS point spectra taken at the indicated locations in the specimen (with the same color labelling the location and the spectrum), which validate the presence of the specific feature associated to the endmember of the corresponding NMF component. **(a)** The plasmon mode around 1.25 eV in component 2 is present in locations of high abundance (red and blue markers) and absent in locations of low abundance (orange and cyan markers). **(b)** The plasmon modes around around 0.75 eV and 1.0 eV in component 3 are present in locations of high abundance (red and orange markers) and absent in locations of low abundance (cyan and blue markers). **(c)** The plasmon mode around 1.75 eV in component 4 is present in locations of high abundance (cyan and blue markers) and absent in locations of low abundance (red and orange markers). Scale bars represent 200 nm

S3 EELS-GDM Electrodynamical Simulations

Electrodynamical simulations of the EELS response of WS_2 nanotriangles for different morphologies were performed using the PyGDM Python package [4, 5] based on the Green Dyadic Method (GDM) to calculate the total electromagnetic field within a nanostructure. The simulations used thickness and side lengths obtained from the structural analysis summarised in Sect. S1 to define the nanostructure volumes. A hexagonal compact mesh was employed for optimal dipole density, with the simulation environment consisting of the nanostructure placed on a Si_3N_4 substrate in vacuum.

S3.1 Edge Dispersions of Nanotriangles

The first simulation series involved an energy window of $[0.5, 2.5]$ eV focusing on plasmonic edge dispersion along the edges of the nanotriangles, as shown in Fig. 3 of the main text. Figs. S11 and S12 present a complete comparison of edges dispersions, showing both experimental data and simulation for corresponding edges.

In Fig. S11, the three edges of the stacked WS_2 nanotriangles are analysed. Due to the small triangle's proximity to the large triangle's edges, thickness influences on plasmonic resonances are observed. Figs. S11a,b show the experimental edge dispersion and simulation of the upper edge, with the latter mirroring Fig. 3b in the main text. Overall, there is good agreement between the experimental dispersion and simulation, with the exception of a 1 eV feature in the experimental data, which is not captured by the simulation. As noted in the main text, this feature originates from the first-order resonance of the small triangle, which "leaks" to the large triangle's edge.

The bottom edge, shown in Figures S11c,d exhibits the significant distortion due to complete overlap between parts of the small triangle and the large triangle's edge. The edge on the right side of the stacked WS_2 nanotriangles, shown in Figures S11e,f, has the small triangle in close proximity to the edge of the large triangle, but without the overlap as seen on the bottom edge.

Although the simulation captures some interesting distortions, many finer details are obscured in the experimental data by the scattering continuum. The 1.25 eV resonance in the edge of the right side of the stacked WS_2 nanotriangles appears diagonally distorted due to thickness differences on each side of the edge. Comparing this with the experimental data, we observe a similar distortion, although leaking of the first-order plasmon mode from the small triangle to the edge of the large triangle may also contribute to the appearance of the distortion, as observed on the other edges.

The edge dispersions of the other nanotriangles are presented in Fig. S12. As the morphologies of these triangles are symmetric, only a single edge was simulated and compared with the experimental data. The experimental edge dispersions show good agreement with the simulated results across all three samples.

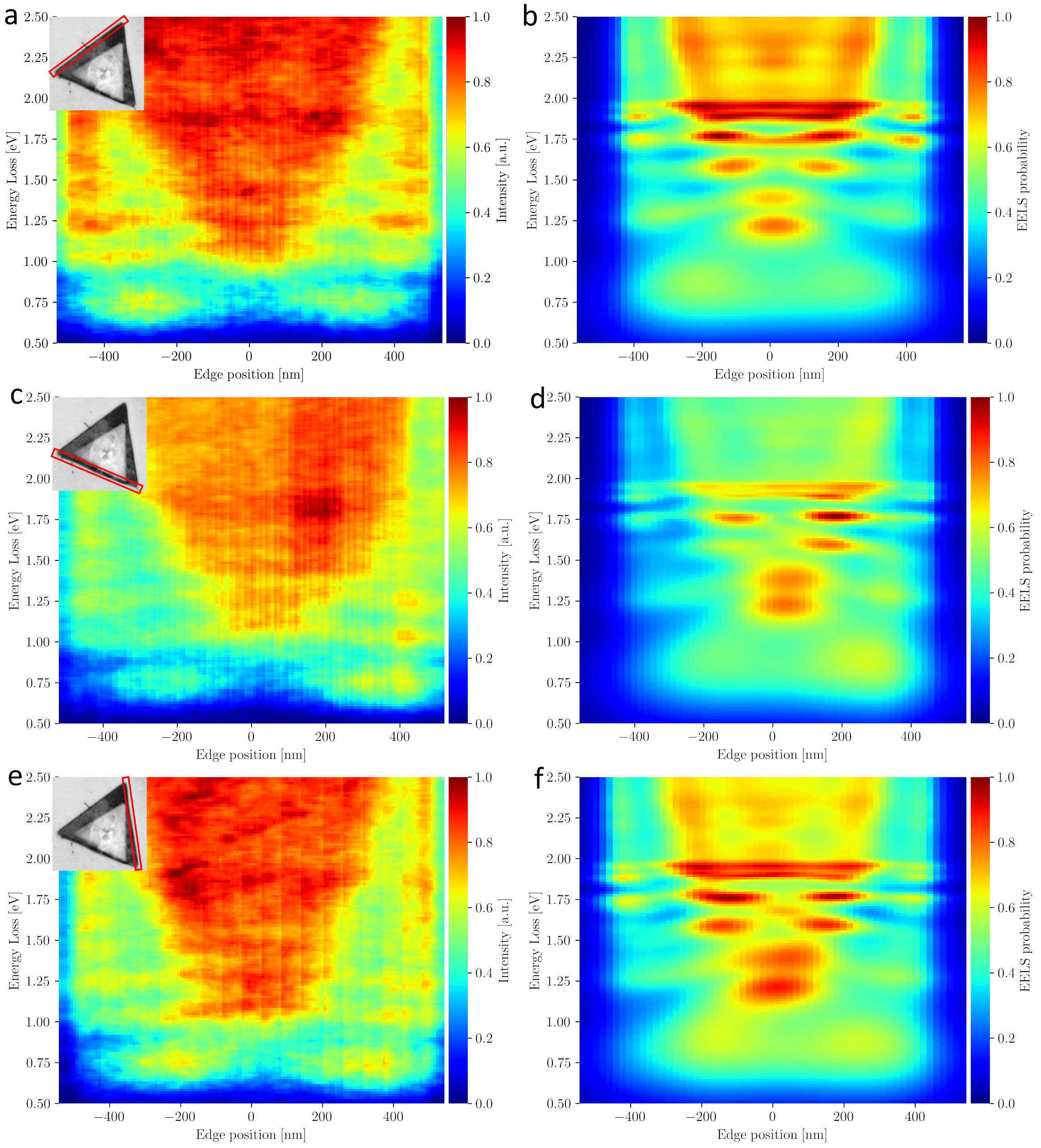

Figure S11: EELS edge profiles of stacked WS_2 nanotriangles. (a,b) Experimental and prediction of the upper edge profile. The experimental data shows a 1 eV feature along the edge originating from the smaller triangle on top, which is not captured in the simulation. The A-exciton at 1.9 eV is clearly visible in both experimental and simulated data, though the detailed split present in the simulation in not observed in the experimental data. **(c,d)** Bottom edge, illustrating thickness effects. The 1.75 eV feature becomes noticeably more intense, as represented in both experimental and simulation data. **(e,f)** Rightmost edge, with parts of the small triangle in close proximity to, but not overlapping with, the egde of the large triangle. This results in a distorted edge profile similar to the upper edge in (a) and (b), with notable differences. The 1 eV resonances from the small triangle appears with greater clarity here. All plots are normalised with their respective maximum values.

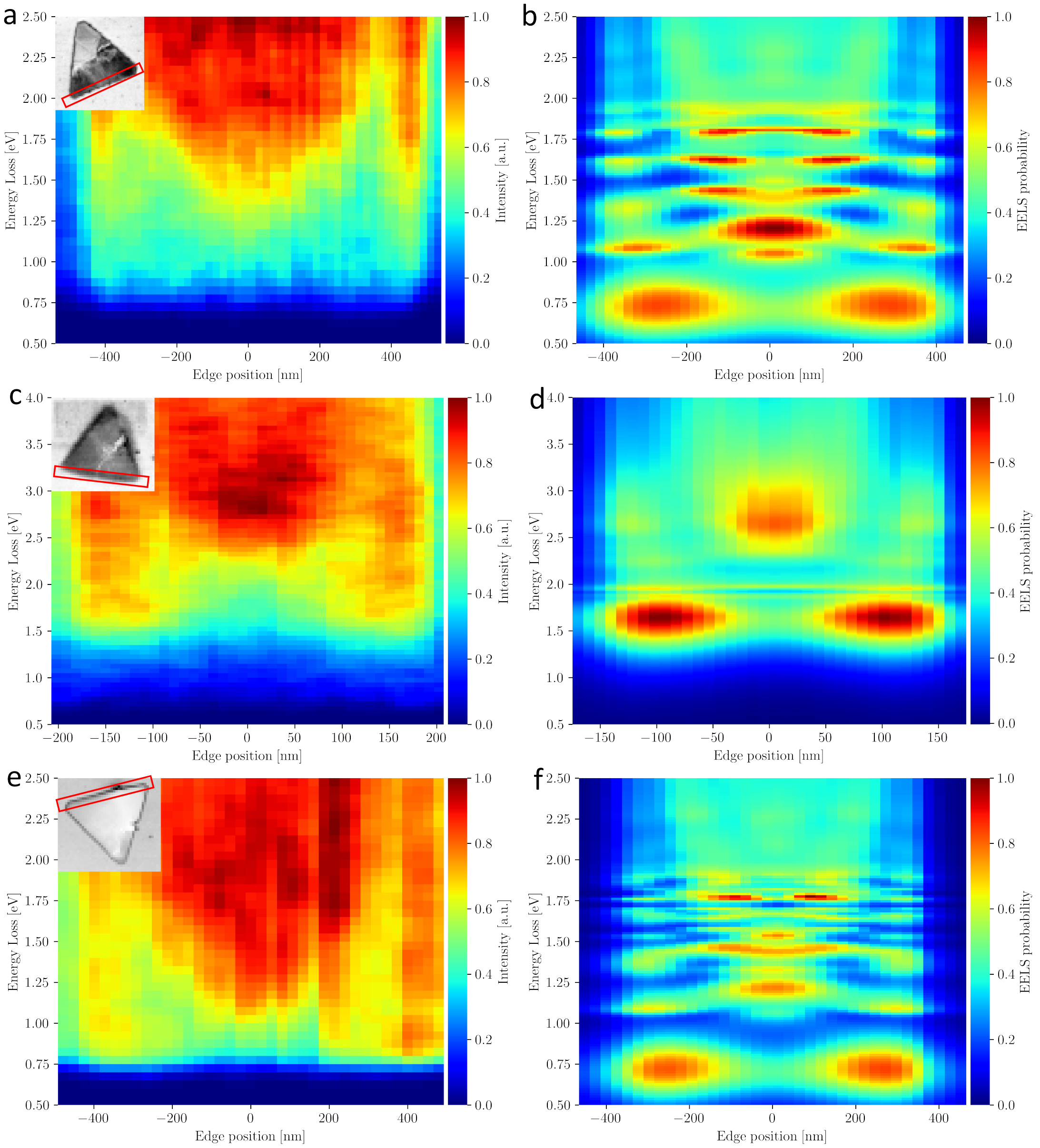

Figure S12: Edge dispersions of the other three triangles. (a,b) Experimental and prediction for the edge profile of the 920 nm triangle. Due to the lower spectral resolution, the experimental data is less detailed than in Fig. S11. **(c,d)** Experimental and prediction for the edge profile of the 330 nm triangle. The two plasmonic resonances observed near 1.75 eV and 2.7 eV in the experimental data correspond well with the simulation. **(e,f)** Experimental and prediction for the edge profile of the 880 nm triangle. All color bars are normalized to their respective maximum values.

S3.2 EELS Probability Maps

Similar to Fig. 4 in the main text, we compare the abundance maps of the additional nanotriangles and their resonances modes with the simulated EELS probability maps.

In Fig. S13d, the second-order resonance shows a high probability at the centre of the triangle, though this feature is not captured in the decomposition-based abundance map shown in Fig. S13b.

In Fig. S14f, the third-order resonance appears notably different from other third-order resonances. Likely due to the increased thickness of the nanotriangle, the central region exhibit a much higher probability. This higher probability is partially reflected in the abundance map in Fig. S14c, albeit at a lower intensity than seen in the simulation.

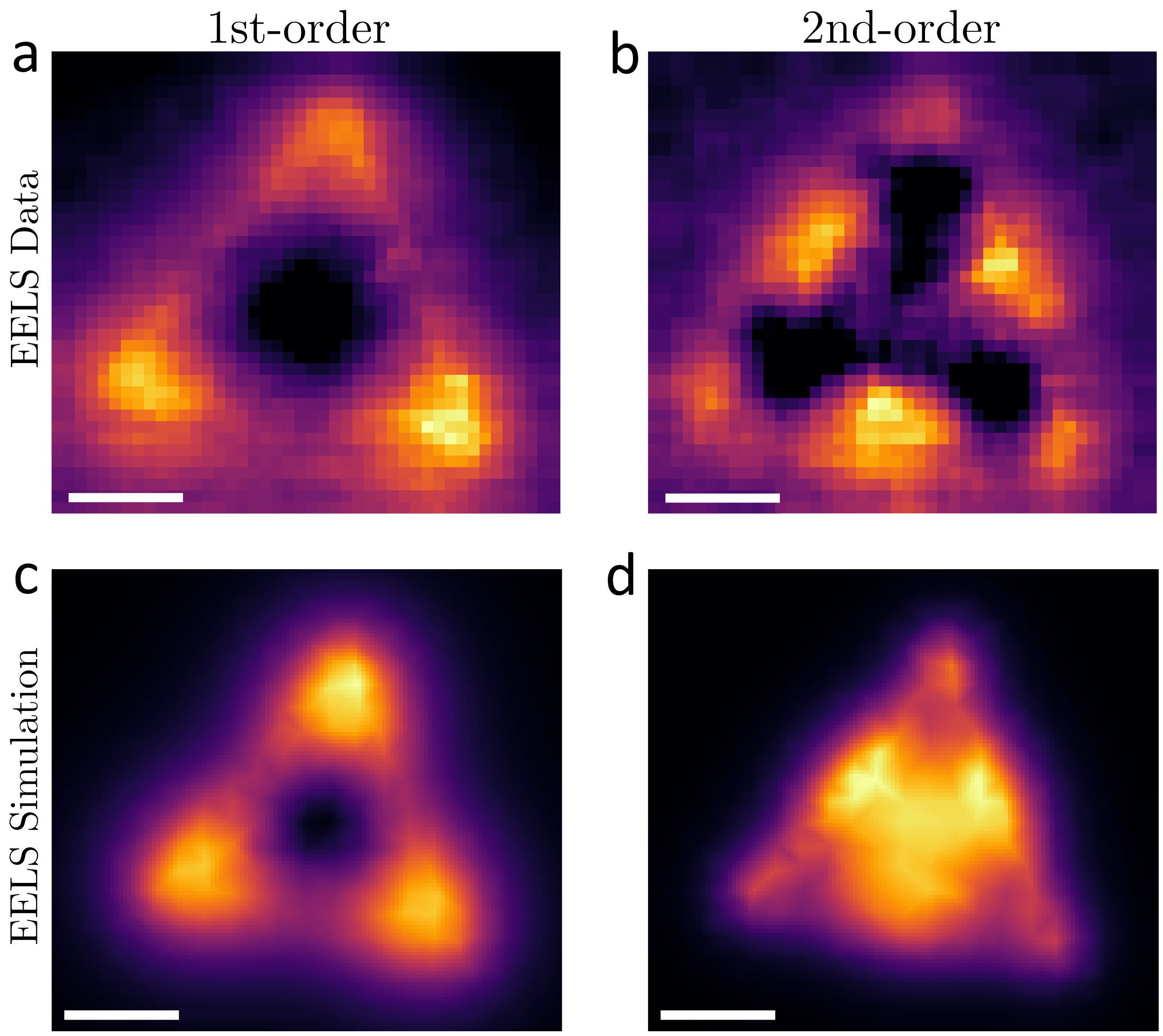

Figure S13: EELS probability maps of localised plasmon resonances in a 330 **nm nanotriangle. (a,b)** Abundance maps of the 330 nm nanotriangle. The energy loss values for the modes derived from the endmembers are: **a** 1.78 eV and **b** 2.68 eV. **(c,d)** Corresponding simulated maps. The simulation intervals are: **c** $[1.65, 1.85]$ eV and **d** $[2.6, 2.8]$ eV. All scale bars are 100 nm.

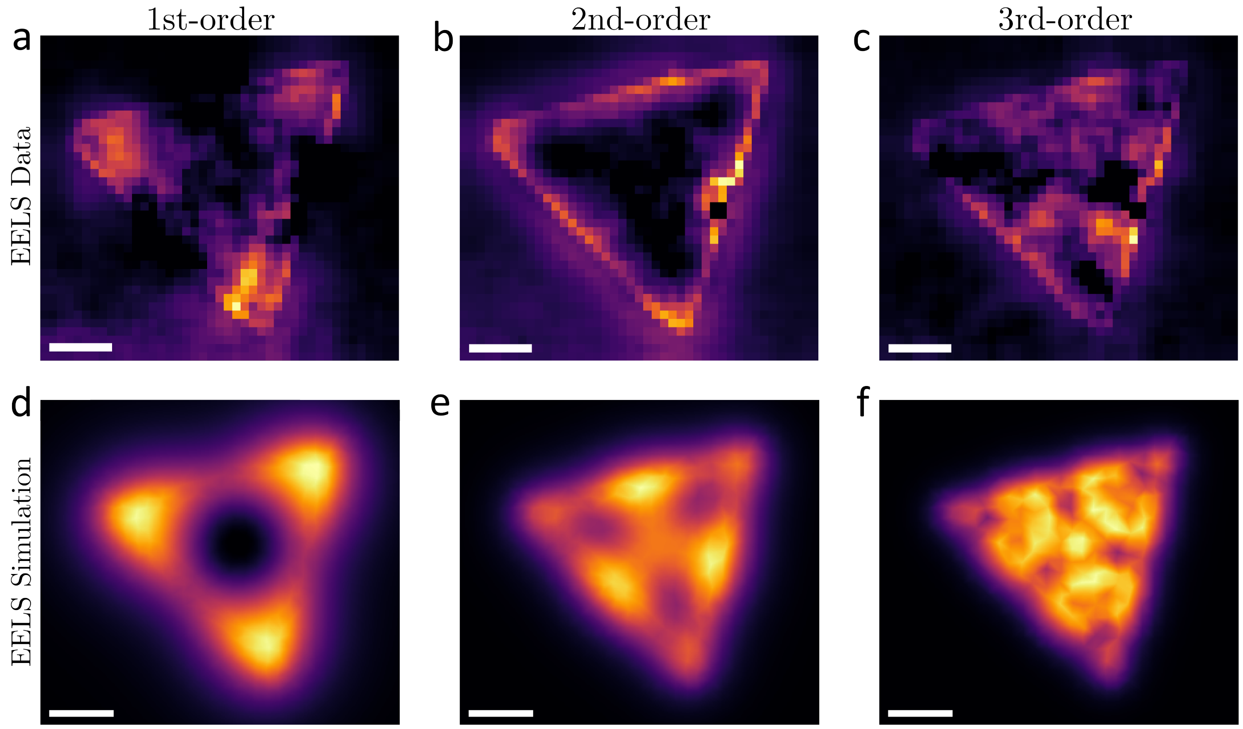

Figure S14: EELS probability maps of localised plasmon resonances in an 880 nm nanotriangle. **(a-c)** Abundance maps of the 880 nm nanotriangle. The energy loss values for the modes obtained from the endmembers are: **a** 0.87 eV, **b** 1.26 eV and **c** 1.69 eV. **(c-e)** Corresponding simulated maps. The simulation intervals are: **d** $[0.7, 0.9]$ eV, **e** $[1.1, 1.3]$ eV and **f** $[1.65, 1.85]$ eV. All scale bars are 200 nm.

S3.3 Potential Limitations of the Simulation Model

Given that the WS_2 nanostructures considered in this work are CVD-grown, small imperfections such as asymmetries or structural defects are naturally present. In the PyGDM simulation model used here, these natural imperfections are not accounted for. This choice is based on the rationale that residual crystal imperfections are not expected to have a qualitative impact on the features being simulated.

While the rasterization and spectral bin sizes in the simulation were matched to the EELS experiments for an accurate one-to-one comparison, microscope conditions such as lens aberrations are not included in the numerical simulation. Specifically, the incoming fast electron is assumed to be perpendicular to the specimen, with an energy of 200 keV and no broadening function applied. Nevertheless, due to our data-driven machine learning approach to remove the zero-loss peak, these simplifications do not introduce any practical issues for interpreting the numerical simulation outcomes in terms of the underlying plasmonic dynamics.

Regarding the mathematical framework underlying the pyGDM simulations, as stated in Wiecha et al. (2022) [5], additional losses described by the so-called *bulk loss probability*, proportional to the

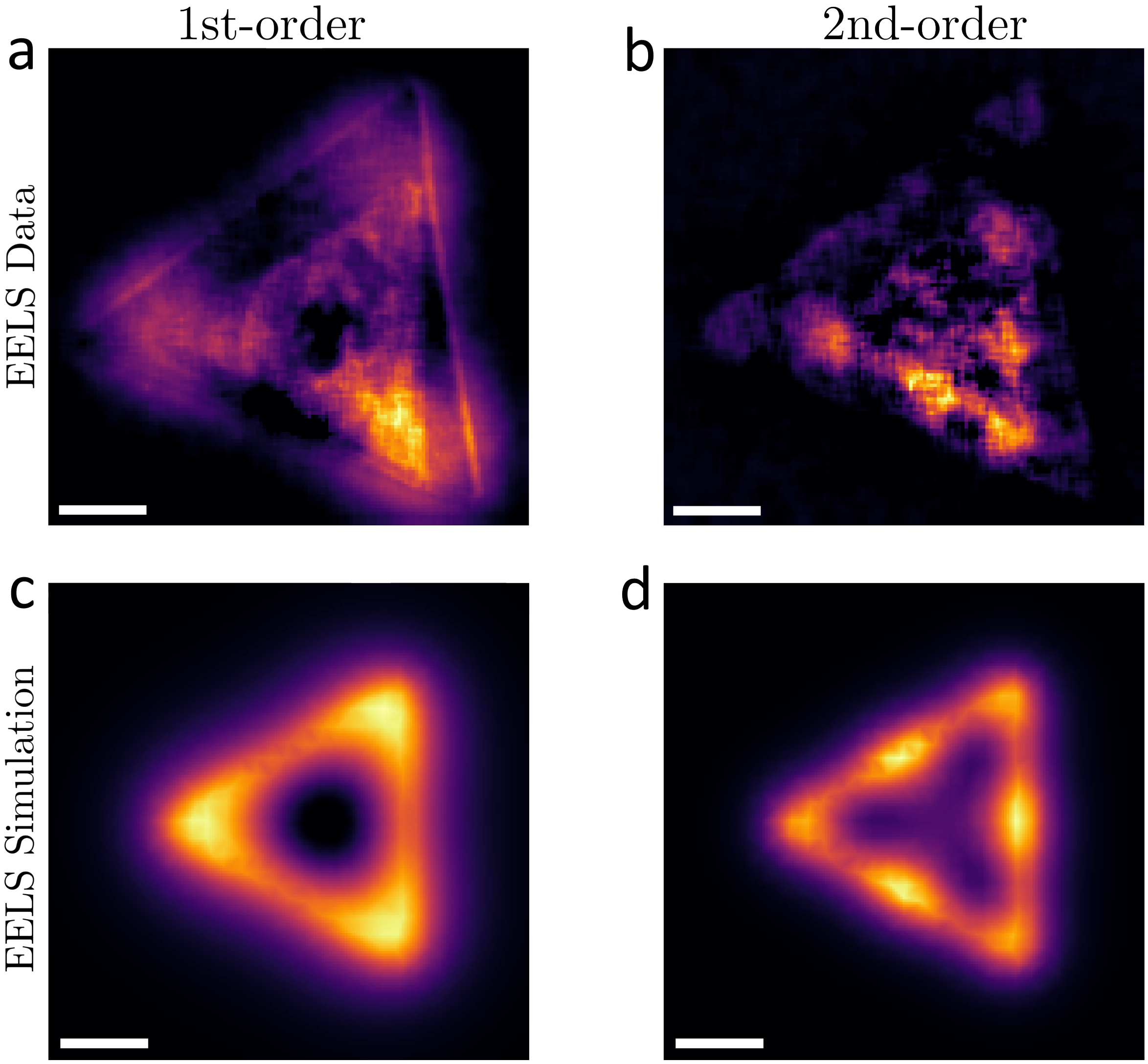

Figure S15: EELS probability maps of localised plasmon resonances in a 680 **nm nanotriangle.** **(a,b)** Abundance maps of the 680 nm nanotriangle. The energy loss values for the modes obtained from the endmembers are: **a** 1.02 eV and **b** 1.54 eV. **(c,d)** Corresponding simulated maps. The simulation intervals are: **c** $[0.9, 1.1]$ eV and **d** $[1.4, 1.6]$ eV. All scale bars are 200 nm.

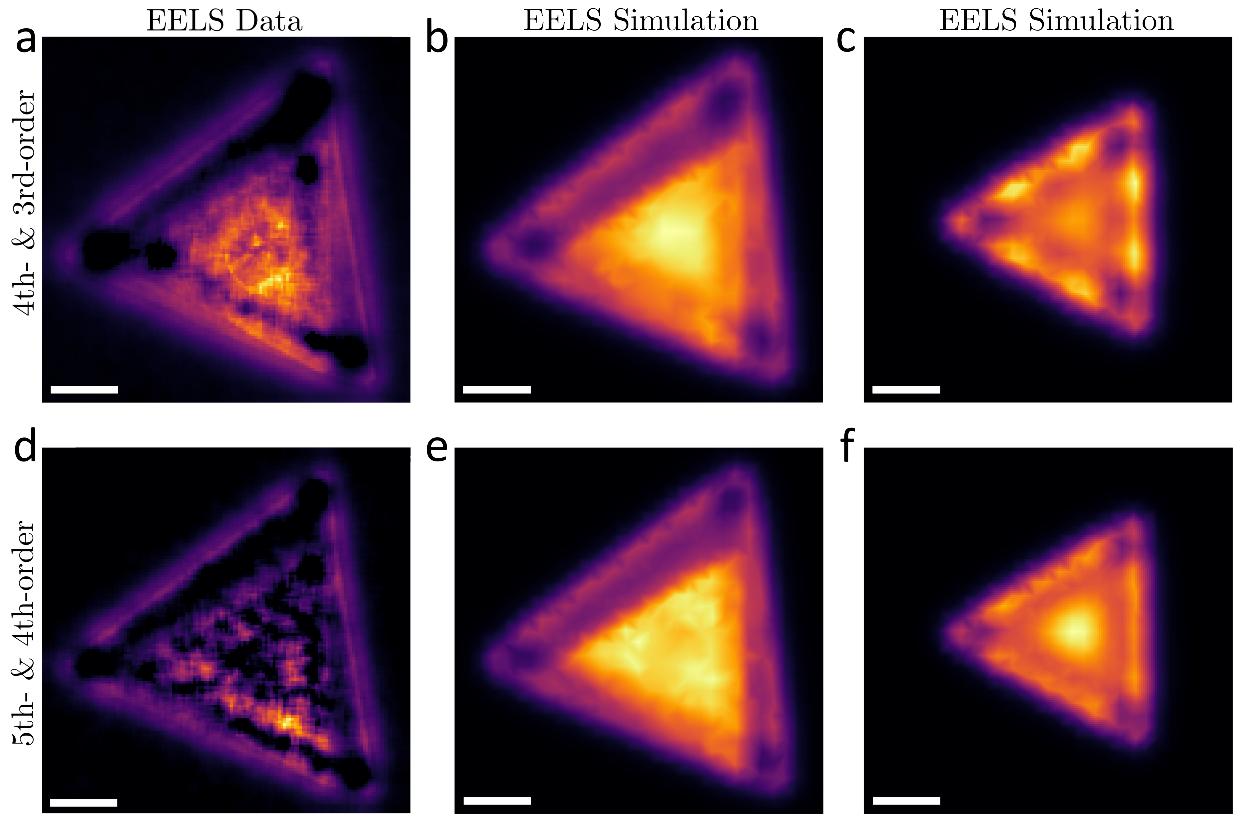

Figure S16: EELS probability maps of higher order localised plasmon resonances in the 680 **nm and** 980 **nm nanotriangle. (a,d)** Abundance maps of the stacked WS_2 nanotriangles, displaying high-order plasmon resonances. The energy loss values for the modes obtained from the endmembers are: **a** 2.09 eV and 2.65 eV. **(b,c,e,f)** Corresponding simulated maps for the modes shown in (a) and (d). The simulation intervals are: **b** [2.05, 2.25] eV, **c** [2.1, 2.3] eV, **e** [2.5, 2.7] eV and **f** [2.6, 2.8] eV. The higher order resonance modes of the small triangle, although present in the same energy range, are most likely mixed with the saturation seen in the simulation maps of the large triangle. As a result, although the abundance maps do show hints along the edges of the resonance modes of the small triangle, the nodes are not distinct enough to extract the edge profiles. All scale bars are 200 nm.

thickness of the traversed material and the imaginary part of $1/\varepsilon(\omega)$, are not included. Given that our specimens are relatively thin and these effects do not significantly influence the edge-localised plasmonic resonances, no qualitative impact on the interpretation of the results is expected.

S4 Extracting Edge Modes in WS_2 nanotriangles from NMF

The values of the energy loss associated to each of the identified edge-localised plasmonic modes are obtained by fitting the endmembers of the NMF components with Gaussians functions, as shown for illustration in Fig. S5.

In order to extract the phase shift and wavenumbers of the edge modes in the WS_2 nanotriangles, we model the system as a linear Fabry-Perot cavity of length L [6], where the incident plasmon wave reflects at the cavity boundary with a phase shift φ.

$$E_i = e^{ik(x-x_0)}\,, \tag{5}$$

$$E_r = |r|e^{ik(2L-x)}e^{i\varphi}\,. \tag{6}$$

Assuming a reflection coefficient $|r| = 1$, the total electric field E_{total} is the sum of the incident and the reflected waves:

$$E_{\text{total}} = e^{ik(x-x_0)} + e^{ik(2L-(x-x_0))}e^{i\varphi}\,. \tag{7}$$

Rewriting E_{total} in the terms of sine and cosine components, we get:

$$E^2_{\text{total}} = [\cos(k(x-x_0)) + \cos(2kL - k(x-x_0) + \varphi)]^2 + [\sin(k(x-x_0)) + \sin(2kL - k(x-x_0) + \varphi)]^2$$

The wavenumber k_n is related to the phase shift φ by the following relation:

$$k_n L = n\pi - \varphi\,. \tag{8}$$

By fitting Eq. (8) to the edge profiles obtained from the NMF abundance map, we can obtain the phase shift φ and thereby calculate the wavenumbers k_n of the associated edge modes.

Fig. 5 in the main manuscript, it was shown how the experimental measurements of the dispersion relations of the plasmonic modes are well described by a quadratic model, as expected from surface plasmon characteristics. In Fig. S17 we add to Fig. 5 also the results of a linear fit. It is clear from the data versus model comparison that the linear fit is insufficient to describe the observed values of the dispersion relations for the resonance modes, further validating its plasmonic interpretation.

The Fabry-Perot interpretation assumed here is thus consistent with the quadratic dispersion that is expected for edge-localised plasmonic resonances [7, 8]. Together with the excellent quantitative agreement between the observed abundance maps and the associated PyGDM electrodynamical simulations, this dispersion relation analysis provides corroborating evidence for the plasmonic nature of the observed modes. We note that WS_2 in particular, and TMDs in general, are known to display metallic-like behaviour along the edges due to exposed transition metal atoms [9, 10, 11, 12]. The resulting metallic-dielectric interface provides favourable conditions for the formation plasmonic resonances, similar to noble metal structures.

Other standing wave phenomena such as Whispering Gallery Modes (WGM) have been observed in both dielectric and metallic nanoparticles [13, 14]. However, WGM require high-rotational

symmetry (e.g. circular or spheric) to appear, which is not present in the nanotriangle geometry under consideration. Furthermore, WGM are not expected to follow a quadratic dispersion relation. Likewise, while Mie resonances often associated to dielectric particles can support multipolar modes, their dispersion relation is not inherently quadratic and also they are typically volumetric and lack strong edge localisation.

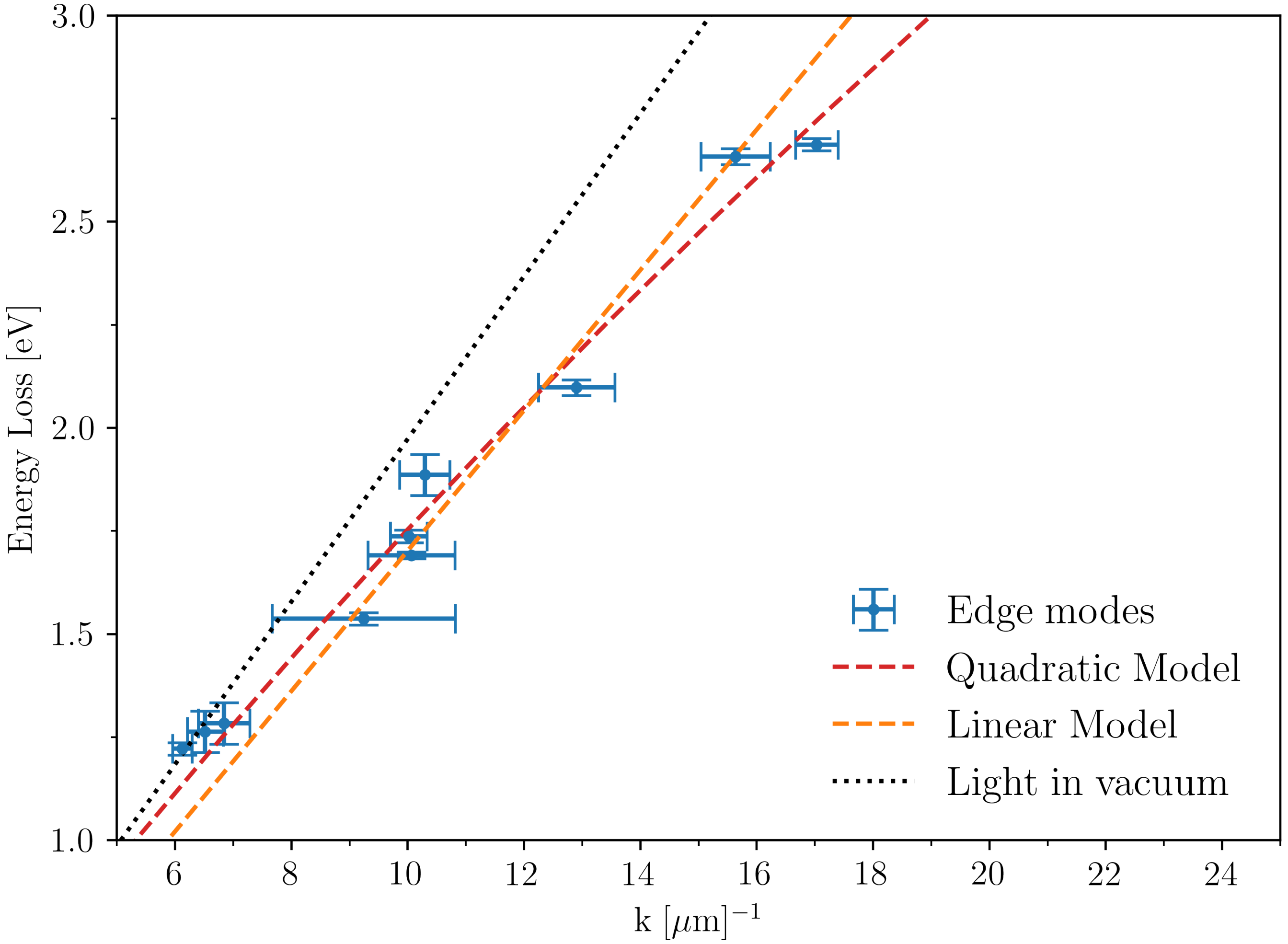

Figure S17: Linear fit to the dispersion relation. Same as Fig. 5 in the main manuscript, now with the addition of a linear model fit. The linear model ($R^2 = 0.95$) describes the experimental measurements of the dispersion relations of the plasmonic modes markedly worse than the default quadratic model ($R^2 = 0.98$) .

S4.1 Advantages of NMF Over Conventional Intensity Mapping Methods

In Fig. S18 we compare the edge mode profile obtained from NMF-derived abundance mapping to that from conventional integrated intensity mapping, using the fifth-order resonance mode of the large triangle as an example. The abundance map from NMF decomposition reveals the modes

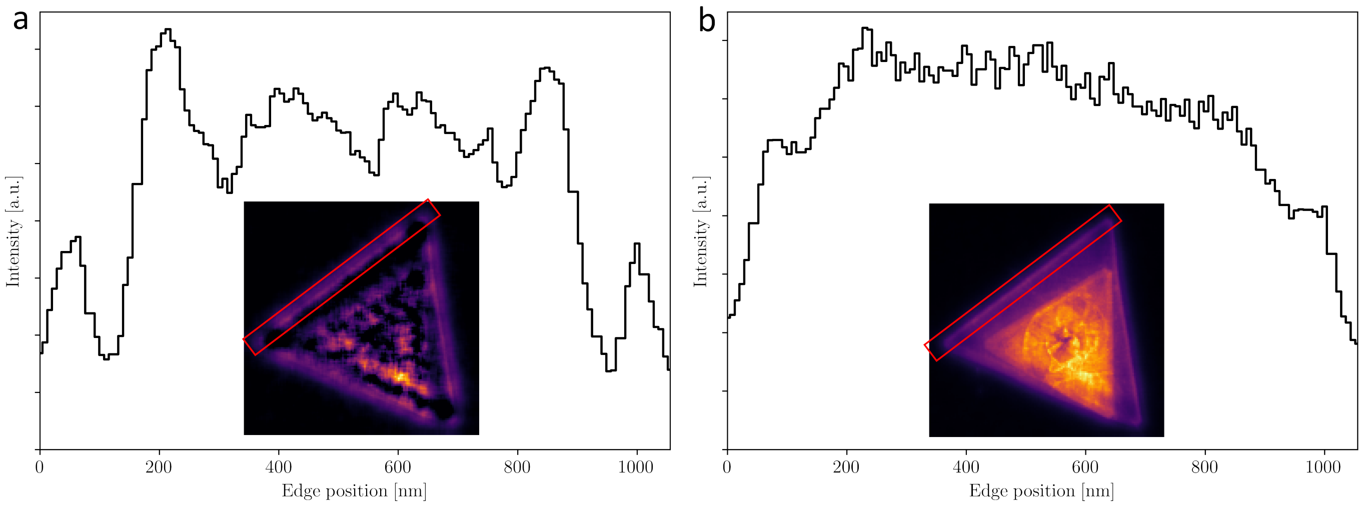

Figure S18: Comparison of NMF-based abundance mapping with integrated intensity mapping. **a)** Edge profile of the fifth-order resonance mode of the large triangle as shown by the abundance map (inset) derived from NMF decomposition. **b)** Edge profile of the same resonance mode obtained by integrating the EELS intensity over the energy window from 2.3 to 2.6 eV where the fifth-order mode is present.

of the resonance mode much more clearly than the integrated intensity method, which does not resolve the nodes as well and make it difficult to fit the profile accurately with Eq. (8). Using NMF decomposition provides unambiguous energy loss values and a well-defined map profile, offering a clearer interpretation of resonance modes and their spatial distribution compared to traditional intensity mapping methods.

References

[1] Iakoubovskii, K., Mitsuishi, K., Nakayama, Y. & Furuya, K. Thickness measurements with electron energy loss spectroscopy. *Microscopy Research and Technique* **71**, 626–631 (2008).

[2] Roest, L. I., van Heijst, S. E., Maduro, L., Rojo, J. & Conesa-Boj, S. Charting the low-loss region in electron energy loss spectroscopy with machine learning. *Ultramicroscopy* **222**, 113202 (2021). URL `https://www.sciencedirect.com/science/article/pii/S0304399121000012`.

[3] Brokkelkamp, A. *et al.* Spatially resolved band gap and dielectric function in two-dimensional materials from electron energy loss spectroscopy. *Journal of Physical Chemistry A* **126**, 1255–1262 (2022).

[4] Wiecha, P. R. pygdm—a python toolkit for full-field electro-dynamical simulations and evolutionary optimization of nanostructures. *Computer Physics Communications* **233**, 167–192 (2018).

[5] Wiecha, P. R. *et al.* "pygdm" - new functionalities and major improvements to the python toolkit for nano-optics full-field simulations. *Computer Physics Communications* **270** (2022).

[6] Campos, A. *et al.* Plasmonic breathing and edge modes in aluminum nanotriangles. *ACS Photonics* **4**, 1257–1263 (2017).

[7] Saito, H. & Kurata, H. Direct measurement of dispersion relation for surface plasmon-polaritons on silver nanoantennas (2014).

[8] Wu, Y. *et al.* Infrared plasmonics: Stem-eels characterization of fabry-pérot resonance damping in gold nanowires. *Physical Review B* **101** (2020).

[9] López-Urías, F. *et al.* Electronic, magnetic, optical, and edge-reactivity properties of semiconducting and metallic ws2 nanoribbons. *2D Materials* **2** (2015).

[10] Skúlason, E. *et al.* Density functional theory calculations for the hydrogen evolution reaction in an electrochemical double layer on the pt(111) electrode. *Physical Chemistry Chemical Physics* **9**, 3241–3250 (2007).

[11] Tinoco, M., Maduro, L. & Conesa-Boj, S. Metallic edge states in zig-zag vertically-oriented mos2 nanowalls. *Scientific Reports* **9**, 15602 (2019). URL `https://doi.org/10.1038/s41598-019-52119-3`.

[12] Lei, W., Xiao, J. L., Liu, H. P., Jia, Q. L. & Zhang, H. J. Tungsten disulfide: synthesis and applications in electrochemical energy storage and conversion (2020).

[13] Hyun, J. K., Couillard, M., Rajendran, P., Liddell, C. M. & Muller, D. A. Measuring far-ultraviolet whispering gallery modes with high energy electrons. *Applied Physics Letters* **93** (2008).

[14] Auad, Y. *et al.* Unveiling the coupling of single metallic nanoparticles to whispering-gallery microcavities. *Nano Letters* **22**, 319–327 (2022).
